# Clinical and epidemiological characteristics of leptospirosis in patients under and over 5 years of age in primary health centers in the Peruvian Amazon, 2022–2024

**DOI:** 10.1371/journal.pntd.0013473

**Published:** 2026-06-25

**Authors:** Stefano V. Davila-Philipps, Tery Vasquez Hassinger, Kary K. Vela-Tello, Jorge I. Carrasco-Celi, Yoki N. Rios-Alava, James L. Vasquez-Lechuga, Marcos H. Parimango-Alvarez, Johan Marin-Lizarraga, Edgar A. Ramirez-García, Karine Zevallos

**Affiliations:** 1 Facultad de Medicina, Universidad Nacional de la Amazonia Peruana, Iquitos, Loreto, Perú; 2 Hospital Regional de Loreto, Iquitos, Loreto, Perú; 3 Universidad Científica del Sur, Lima, Lima, Perú; Cornell University, UNITED STATES OF AMERICA

## Abstract

**Background:**

Leptospirosis is an emerging zoonotic disease with high incidence in tropical regions such as the Peruvian Amazon. Although it affects individuals of all ages, knowledge about its clinical and epidemiological profile in children under 5 years remains limited. In this age group, leptospirosis may present with nonspecific manifestations, which may hinder timely recognition in primary care.

**Methodology:**

We conducted a retrospective cross-sectional study using secondary data from medical records and epidemiological forms of MAT-confirmed leptospirosis cases evaluated between 2022 and 2024 at four primary health care centers in Belén, Loreto, Peru. A total of 2,325 patients with clinical suspicion of leptospirosis were evaluated during the study period, of whom 400 MAT-confirmed cases with sufficient information for analysis were included. Comparisons between patients under 5 years and those aged 5 years or older were exploratory.

**Results:**

Among the 400 confirmed cases, 28 occurred in children under 5 years. In this age group, the most frequently recorded symptoms were malaise (78.6%) and fever (71.4%). Half of the children under 5 years lived in flood-prone areas, and 46.4% were evaluated within the first 3 days after symptom onset. Compared with older patients, children under 5 years more often showed nonspecific symptom profiles, whereas headaches and chills were less frequently recorded. Nutritional classification and selected symptom patterns also differed between age groups, although these comparisons were exploratory and based on a small pediatric sample.

**Conclusion:**

In this series of MAT-confirmed leptospirosis cases managed at the primary health care centers, children under 5 years commonly presented with nonspecific symptoms such as malaise and fever. In an endemic Amazonian setting where multiple febrile illnesses coexist, this pattern may hinder early recognition. These findings support the need to strengthen clinical suspicion, diagnostic capacity, and epidemiological surveillance for pediatric leptospirosis in primary care. Further studies with larger pediatric samples are needed to better characterize age-related differences in presentation.

## Author summary

Leptospirosis is an infection acquired through contact with water or soil contaminated with the urine of infected animals, and it is especially common in tropical settings such as the Peruvian Amazon. Although it can affect people of all ages, little is known about how it presents in children under 5 years. In this study, we analyzed 400 MAT-confirmed leptospirosis cases identified at primary health care centers in Belén, one of the most socially and environmentally vulnerable districts in the Loreto region of Peru. Among the 28 children under 5 years included in the study, the most frequently recorded symptoms were malaise and fever, and their presentation tended to be less specific than that of older patients. Many of these children were identified in flood-prone settings, and some differences were also observed in nutritional classification and timing of consultation. Because the pediatric sample was small and the study was based on retrospective primary-care records, these findings are best understood as descriptive. Still, they suggest that mild leptospirosis in young children may be underrecognized in endemic areas where other febrile illnesses, such as dengue, malaria, and arboviral infections, also circulate. Improving clinical suspicion, surveillance, and diagnostic capacity at the primary health care centers may support earlier recognition and care in this age group.

## Introduction

Leptospirosis is a globally distributed zoonotic disease caused by spirochete bacteria of the genus Leptospira, with a wide clinical spectrum ranging from subclinical infection to severe disease with multiorgan failure. Transmission to humans occurs through direct or indirect exposure to urine from infected wild or domestic animals, particularly rodents, dogs, pigs, and cattle. In reservoir hosts, Leptospira colonizes the proximal renal tubules and is excreted in urine, contaminating soil and water. In settings with inadequate sanitation, frequent flooding, and precarious housing, these environmental conditions favor bacterial persistence and increase the risk of human exposure [1,2].

Leptospira has worldwide distribution, with the highest incidence in tropical and subtropical regions, where it contributes substantially to febrile illness morbidity and mortality. Regions with the greatest estimated burden include Sub-Saharan Africa, Latin America and the Caribbean, South and Southeast Asia, and Oceania. Global incidence exceeds one million cases annually, with approximately 60,000 deaths [3]. In the Peruvian Amazon, leptospirosis is endemic, with epidemic surges occurring after periods of heavy rainfall and flooding. In Loreto, outbreaks and increased transmission have been documented in association with Amazon River overflows, and national and regional surveillance in 2023 reported a marked increase in cases in Loreto during the post-rainy season, including outbreak investigations in affected localities. El Niño-related rainfall and flooding events in Peru have likewise been associated with increased leptospirosis transmission [4,5]. As of epidemiological week 52 of 2024, Peru had reported 9,520 leptospirosis cases, an incidence of 27.91 per 100,000 inhabitants, and 16 confirmed deaths, with a substantial proportion concentrated in high-burden Amazonian departments, particularly Loreto, Ayacucho, Madre de Dios, and Ucayali [6]. In this high-burden setting, where many suspected cases are initially assessed outpatient primary-care settings, understanding age-stratified clinical profiles is relevant to improve early clinical suspicion, guide targeted diagnostic testing, and support timely treatment [7].

Age has been associated with clinically meaningful differences in leptospirosis presentation and outcomes. Early manifestations are often nonspecific and overlap with other causes of acute febrile illness in tropical settings, including dengue, malaria, rickettsial infections, and bacterial sepsis, which may delay recognition and appropriate management. Defining age-stratified clinical profiles may therefore help strengthen early clinical suspicion in endemic areas [8–11]. Classic severe manifestations may be less frequently observed in pediatric patients than in adults, but they may also present with more nonspecific or atypical patterns that are harder to recognize in primary health care centers [12,13]. In early childhood, clinical assessment may be complicated by limited self-reporting of subjective symptoms, and children with leptospirosis may less frequently exhibit the classic severe features seen in adults, such as jaundice and overt renal dysfunction, thereby reducing early diagnostic suspicion at the first point of care [13–16].

Belén, a district of Maynas Province in Loreto, experiences seasonal river flooding, inadequate sanitation, precarious housing, and limited access to safe water, all of which create favorable conditions for leptospirosis transmission. These environmental and social conditions make Belén a priority area for leptospirosis surveillance and public health intervention.

Although leptospirosis has been extensively described in general populations, relatively few studies have systematically compared clinical and epidemiological differences across age groups, particularly in outpatient or primary health care centers and among children under 5 years of age. This gap is especially relevant in the Peruvian Amazon, where early presentations frequently overlap with other causes of acute febrile illness and where most suspected cases are managed initially at the first level of care. Therefore, this study aimed to describe and compare the clinical manifestations and epidemiological characteristics of confirmed leptospirosis cases in patients under 5 years and those aged 5 years or older evaluated at four primary health care centers in Belén, Loreto. By defining age-stratified outpatient profiles in this setting, the findings may inform prevention strategies and support diagnostic suspicion and initial clinical decision-making at the primary health care centers.

## Methods

### Ethics statement

The study was approved by the Ethics Committee of the Regional Hospital of Loreto “Felipe Santiago Arriola Iglesias” (No. 091-CIE-HRL-2024). This was a retrospective study based on routinely collected clinical records. All data were de-identified prior to analysis and were handled in accordance with institutional policies to protect confidentiality, with access restricted to the study team. The ethics committee granted a waiver of informed consent because the study used existing medical records, posed minimal risk to participants, and did not involve direct contact with patients.

### Study design

This was a retrospective cross-sectional study using secondary data from clinical records of patients with Microscopic Agglutination Test (MAT) confirmed leptospirosis treated at four primary health care centers in the district of Belén, Maynas Province, Loreto region, Peru, between January 1, 2022, and December 31, 2024. The study was designed as a descriptive and exploratory analysis; therefore, no a priori confirmatory hypothesis was tested.

### Study setting

**Fig 1:**
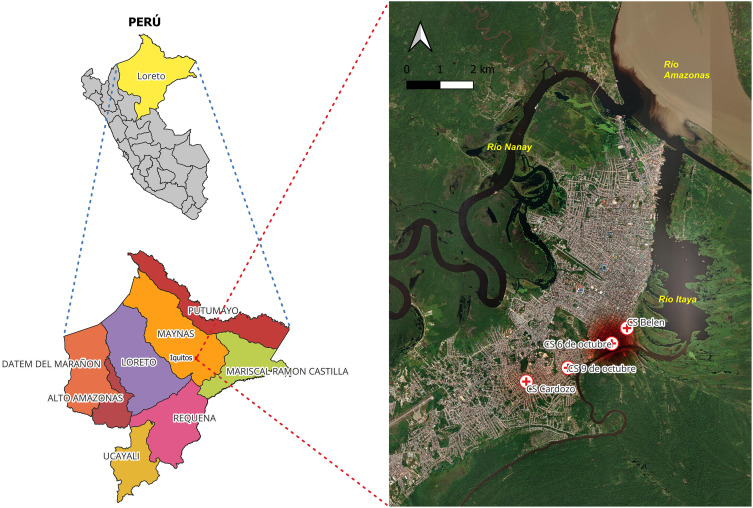
Geographic location of the study area and included primary health centers in Loreto, Peru.

The study was conducted in four first-level Ministry of Health primary health care centers serving Belén and nearby urban and urban-marginal sectors of Iquitos: 6 de Octubre health center, 9 de Octubre health center, Cardozo health center, and Belén health center (Fig 1). These facilities provide primary care in areas characterized by high population density, frequent flooding, and environmental conditions favorable to tropical infectious diseases. 6 de Octubre health center is a category I-3 health center that also provides 24-hour emergency services and participates in epidemiological surveillance, whereas the other participating centers provide routine first-level care, generally with more limited emergency coverage. These differences in service organization and catchment characteristics should be considered when interpreting the distribution of confirmed cases across centers.

Belén is a district of the province of Maynas, Loreto region, in the northeastern Peruvian Amazon. It is among the most socially and environmentally vulnerable urban areas of Iquitos, with an estimated population of approximately 76,000 inhabitants, around 75% of whom live in flood-prone areas, particularly in the lower part of the district along the Itaya River. The area has a warm, humid, rainy tropical climate, with temperatures ranging from 17°C to 38°C and annual rainfall exceeding 2,600 mm [17]. During seasonal river flooding, many households become partially submerged, creating unsanitary conditions due to water stagnation, inadequate waste disposal and limited sanitation infrastructure. These conditions increase exposure to contaminated water and other environmental hazards, facilitating the transmission of infectious diseases. Children under 5 years of age in Loreto also bear a high burden of other common conditions, including anemia, acute respiratory infections, diarrheal disease, and parasitic infections [18–20]. In this context, the environmental and social conditions of Belén, including seasonal flooding, limited access to potable water, inadequate sanitation, and precarious housing, may facilitate leptospirosis transmission and support the need for surveillance and public health interventions.

### Population and sample

**Fig 2:**
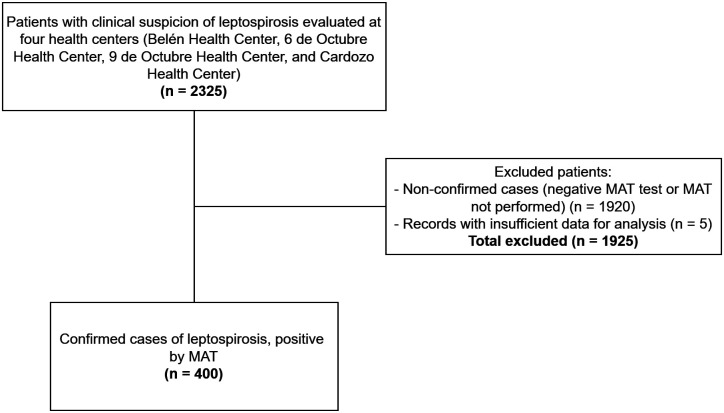
Flow diagram of the inclusion and exclusion of patients with suspected leptospirosis evaluated at four primary health centers.

The study population consisted of patients evaluated for suspected leptospirosis at four primary health care centers in Belén during the study period. A total of 2,325 patients with clinical suspicion of leptospirosis were identified. Of these, 1,920 were excluded because leptospirosis was not confirmed by MAT, either due to a negative result or because the test was not performed, and 5 additional records were excluded because they contained insufficient information for analysis. The final analytical sample included 400 patients with MAT-confirmed leptospirosis (Fig 2). No formal sample size calculation was performed because the study included all eligible MAT-confirmed cases with sufficient information during the study period. Therefore, subgroup comparisons, particularly those involving children under 5 years of age, should be interpreted as exploratory.

Patients were eligible for inclusion if they had a MAT-confirmed diagnosis of leptospirosis and sufficient information in the medical records and epidemiological forms for the variables included in the analysis. Confirmatory diagnosis was based on MAT performed at the National Institute of Health laboratory in Lima. A case was considered confirmed when a single serum sample showed a MAT titer of at least 1:800, with serogroup reactivity recorded. This threshold was selected in accordance with the Peruvian National Technical Health Standard for leptospirosis, which indicates that, when paired sera are not available, a single MAT titer ≥1:800 may confirm the diagnosis in the Peruvian Amazon [21]. Given the retrospective nature of this primary care study, paired acute and convalescent samples were not routinely available for all patients.

### Procedure and data collection

Data were collected from medical records of patients with confirmed leptospirosis and from the Clinical-Epidemiological Research Form for Leptospirosis included in the Technical Health Standard for the Comprehensive Care of People Affected by Leptospirosis. Extracted variables included sociodemographic, clinical, nutritional, laboratory, and treatment-related information.

Patients were categorized into two age groups: under 5 years and 5 years or older. Sociodemographic variables included sex, district of origin, type of area of residence (rural, urban, or urban-marginal), and residence in a flood-prone area. Flood-prone residence was defined based on self-report recorded in clinical or epidemiological forms and referred to living in an area affected by rainfall-related flooding and/or seasonal river overflow.

Clinical variables included recorded signs and symptoms at presentation and time from symptom onset to consultation at the primary health center. Treatment variables included antibiotics, antipyretics, intravenous hydration, and other medications recorded in the chart. Laboratory variables included MAT serogroup reactivity.

Nutritional status was classified using age-standardized anthropometric criteria in accordance with World Health Organization recommendations. In children under 5 years of age, undernutrition was defined as a weight-for-height Z score (WHZ) below −2 standard deviations. In children and adolescents aged 5–18 years, undernutrition was defined as a body mass index-for-age Z score (BAZ) below −2. In adults aged 19 years or older, undernutrition was defined as a body mass index (BMI) <18.5 kg/m² [22,23].

### Statistical analysis

Data were entered into Microsoft Excel (Windows 10 version) and analyzed using IBM SPSS Statistics version 27.0 (Windows 11). Descriptive statistics were used to summarize sociodemographic, clinical, and epidemiological variables. Categorical variables are presented as absolute and relative frequencies (n, %). Clinical and epidemiological variables were analyzed as categorical variables. Time from symptom onset to consultation was summarized using predefined categories and, for bivariate comparisons, was additionally dichotomized as ≤3 versus >3 days. Comparisons between age groups (<5 vs ≥ 5 years) were conducted using Fisher’s exact test because several variables had small cell counts. Variables included in the bivariate analysis were selected based on clinical relevance and frequency observed in the descriptive analysis. All bivariate comparisons, including variables not shown in the main text, are presented in S2 Table. Given that these analyses were exploratory and multiple variables were assessed, no formal correction for multiple testing was applied. Accordingly, p-values were interpreted as descriptive measures of statistical evidence rather than confirmatory tests of association, and no causal or definitive age-related inferences were made. Exploratory bivariate comparisons were retained to identify potential age-related clinical patterns that may inform future analytical studies. Records with insufficient information for the variables included in the analysis were excluded before statistical analysis. All tests were two-sided, and p < 0.05 was used as the nominal threshold for statistical significance.

### Spatial analysis

A descriptive spatial analysis was conducted using geographic information systems (GIS). A reference map was created to contextualize the study area within the Loreto region and Peru. The spatial distribution of confirmed leptospirosis cases was visualized using a Kernel Density Estimation (KDE) map based on the number of confirmed cases recorded at the four participating primary health centers, using a quartic kernel and a 500 m search radius. Thus, the density surface reflects the concentration of confirmed cases by reporting health center rather than patients’ area of residence. Participating health centers were represented as geographic reference points. Base cartography was obtained from OpenStreetMap, and satellite imagery from Esri World Imagery. Spatial processing was performed in QGIS version 3.44.7 using the projected coordinate system UTM Zone 18S.

## Results

**Fig 3:**
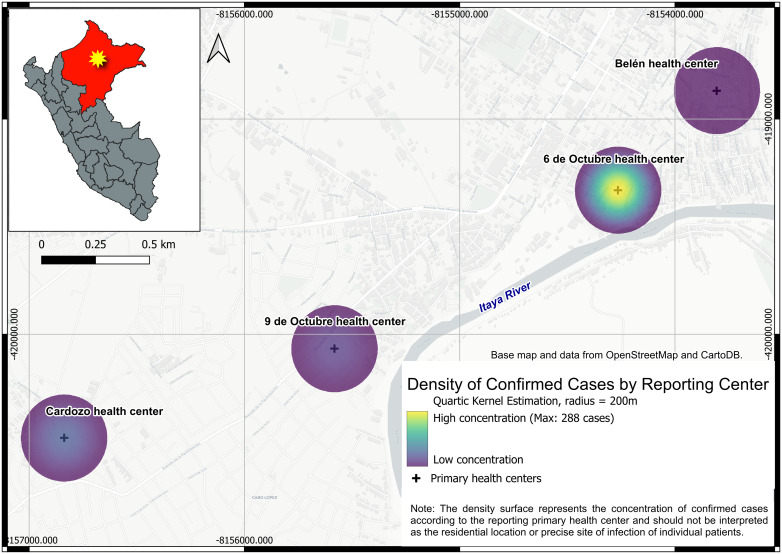
Kernel density map of MAT-confirmed leptospirosis cases reported in primary health centers in the Peruvian Amazon, 2022–2024.

**Fig 4:**
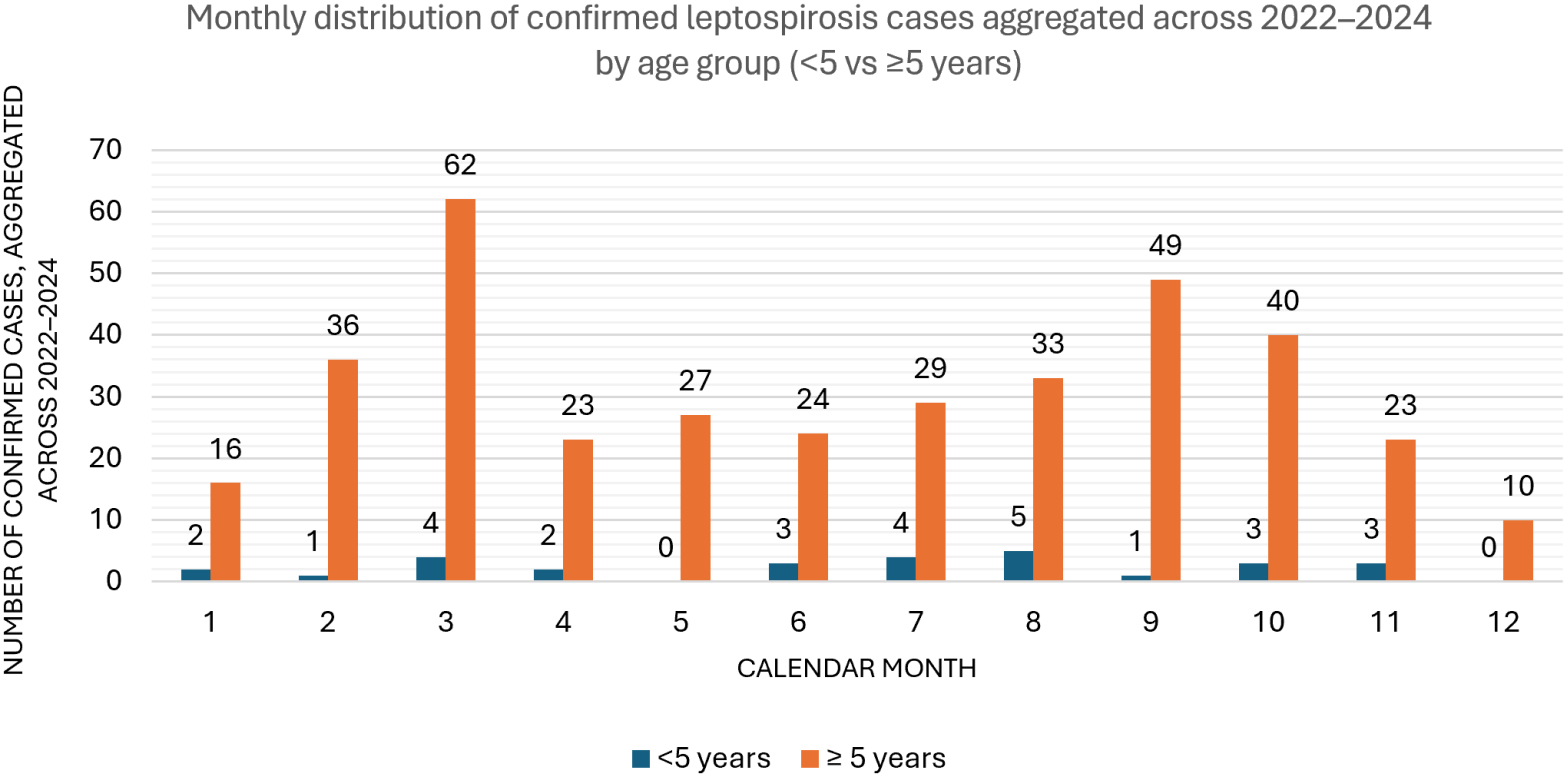
Monthly distribution of confirmed leptospirosis cases by age group (<5 vs ≥5 years) in primary health centers in the Peruvian Amazon, 2022–2024.

There were 81 cases in 2022 (20.3%), 105 cases in 2023 (26.3%), and 214 cases in 2024 (53.5%) (Table 1). Thus, a higher number of confirmed cases were identified in records in 2024 than in prior years. However, as this was a retrospective review and the number of suspected cases evaluated was not available by year, these differences should not be interpreted as annual changes in incidence. Among children under 5 years, most confirmed cases were recorded in 2024 (20/28, 71.4%), whereas among patients aged 5 years or older, 194/372 (52.2%) were recorded in the same year. Cases were unevenly distributed across primary health centers: 6 de Octubre accounted for 288 of 400 confirmed cases (72.0%), followed by Cardozo with 71 (17.8%), 9 de Octubre with 37 (9.2%), and Belén with 4 (1.0%) (Table 1; Fig 3). Center-stratified demographic and clinical characteristics are presented in S1 Table. When monthly counts were aggregated across 2022–2024, patients aged 5 years or older showed the highest numbers of confirmed cases in March (n = 62), September (n = 49), and October (n = 40). Among children under 5 years, the total number of confirmed cases was small (n = 28), with low monthly counts throughout the year and a maximum of 5 cases in August (Fig 4).

**Table 1:** Epidemiological characteristics of patients with confirmed leptospirosis evaluated at primary health centers in the Peruvian Amazon, 2022–2024.

Characteristics	<5 years (n = 28)		≥5 years (n = 372)		Total N = 400	
	Frequency	Percentage	Frequency	Percentage	Frequency	Percentage
Cases reported in 2022	0	0.0%	81	21.8%	81	20.3%
Cases reported in 2023	8	28.6%	97	26.1%	105	26.3%
Cases reported in 2024	20	71.4%	194	52.2%	214	53.5%
Sex						
Male	10	35.7%	173	46.5%	183	45.8%
Female	18	64.3%	199	53.5%	217	54.3%
District of origin						
Belén	27	96.4%	361	97.0%	388	97.0%
San Juan	0	0.0%	9	2.4%	9	2.3%
Iquitos	1	3.6%	2	0.5%	3	0.8%
Health Center						
6 de octubre	21	75.0%	267	71.8%	288	72.0%
Cardozo	4	14.3%	67	18.0%	71	17.8%
Belén	1	3.6%	3	0.8%	4	1.0%
9 de octubre	2	7.1%	35	9.4%	37	9.2%
Area of origin						
Urban	11	39.3%	163	43.8%	174	43.5%
Urban-marginal	5	17.9%	114	30.6%	119	29.8%
Rural	12	42.9%	95	25.5%	107	26.8%
Residence in flood-prone area						
Yes	14	50.0%	160	43.0%	174	43.5%
No	14	50.0%	212	57.0%	226	56.5%

Among children under 5 years, 12 (42.9%) were from rural areas, 11 (39.3%) from urban areas, and 5 (17.9%) from urban-marginal areas. Among patients aged 5 years or older, urban residence predominated (163 cases, 43.8%), followed by urban-marginal (114, 30.6%) and rural areas (95, 25.5%). Residence in flood-prone areas was reported in 14 children under 5 years (50.0%) and 160 patients aged 5 years or older (43.0%). Females accounted for 18 of 28 cases (64.3%) among children under 5 years and 199 of 372 cases (53.5%) among patients aged 5 years or older (Table 1). Overall, the most frequent interval from symptom onset to consultation was 3–6 days (183 cases, 45.8%). Among children under 5 years, the largest proportion presented within 0–3 days (13/28, 46.4%), whereas among patients aged 5 years or older, the largest proportion presented within 3–6 days (171/372, 46.0%) (Table 2).

**Table 2:** Time from symptom onset to consultation among confirmed leptospirosis cases by age group (<5 vs ≥5 years) in primary health centers in the Peruvian Amazon, 2022–2024.

Characteristics	<5 years (n = 28)		≥5 years (n = 372)		Total (n = 400)	
	Frequency	Percentage	Frequency	Percentage	Frequency	Percentage
**Duration of illness**						
0–3 days	13	46.4%	97	26.1%	110	27.5%
3–6 days	12	42.9%	171	46.0%	183	45.8%
More than 6 days	3	10.7%	104	28.0%	107	26.8%

Regarding clinical presentation, the most frequently recorded symptoms in children under 5 years were malaise (22/28, 78.6%) and fever (20/28, 71.4%). Among patients aged 5 years or older, the most frequently recorded symptoms were fever (311/372, 83.6%), headache (247/372, 66.4%), malaise (190/372, 51.1%), and chills (133/372, 35.8%). Other symptoms more commonly recorded in patients aged 5 years or older included nausea/vomiting (23.1%), dizziness (22.6%), myalgia (16.4%), and musculoskeletal pain (10.2%) (Table 3).

**Table 3:** Distribution of clinical symptoms among confirmed leptospirosis cases by age group (<5 vs ≥ 5 years) in primary health centers in the Peruvian Amazon, 2022–2024.

Symptoms	<5 years (n = 28)		≥5 years (n = 372)		Total (n = 400)	
	Frequency	Percentage	Frequency	Percentage	Frequency	Percentage
Fever	20	71.4%	311	83.6%	331	82.8%
Headache	10	35.7%	247	66.4%	257	64.3%
General malaise	22	78.6%	190	51.1%	212	53.0%
Chills	2	7.1%	133	35.8%	135	33.8%
Nausea/Vomiting	6	21.4%	86	23.1%	92	23.0%
Dizziness	2	7.1%	84	22.6%	86	21.5%
Myalgias	4	14.3%	61	16.4%	65	16.3%
Low back pain	2	7.1%	60	16.1%	62	15.5%
Cough	6	21.4%	47	12.6%	53	13.3%
Anorexia	3	10.7%	48	12.9%	51	12.8%
Diarrhea	4	14.3%	44	11.8%	48	12.0%
Abdominal pain	3	10.7%	45	12.1%	48	12.0%
Arthralgias	2	7.1%	37	9.9%	39	9.8%
Musculoskeletal pain	1	3.6%	38	10.2%	39	9.8%
Asthenia	2	7.1%	36	9.7%	38	9.5%
Calf pain	0	0.0%	29	7.8%	29	7.3%
Retro-ocular pain	1	3.6%	28	7.5%	29	7.3%
Rash	2	7.1%	14	3.8%	16	4.0%
Dyspnea	0	0.0%	14	3.8%	14	3.5%
Constipation	1	3.6%	6	1.6%	7	1.8%
Precordial pain (chest pain)	0	0.0%	5	1.3%	5	1.3%
Oliguria	1	3.6%	3	0.8%	4	1.0%
Petechiae	0	0.0%	4	1.1%	4	1.0%
Hematuria	0	0.0%	2	0.5%	2	0.5%
Epistaxis	0	0.0%	2	0.5%	2	0.5%
Gastrointestinal bleeding	0	0.0%	1	0.3%	1	0.3%

The predominant MAT serogroup reactivity in both age groups was Varillal, detected in 24 of 28 children under 5 years (85.7%) and 316 of 372 patients aged 5 years or older (84.9%). Panama reactivity was identified in 2 children under 5 years (7.1%) and 5 patients aged 5 years or older (1.3%), whereas Hurstbridge reactivity was recorded only among patients aged 5 years or older (47 cases, 12.6%). Overall, 317 patients (79.3%) showed reactivity to a single serogroup, while 83 (20.8%) showed reactivity to two or more serogroups (Table 4).

**Table 4:** Distribution of MAT serogroup reactivity among confirmed leptospirosis cases by age group (<5 vs ≥ 5 years) in primary health centers in the Peruvian Amazon, 2022–2024.

Characteristics	<5 years (n = 28)		≥5 years (n = 372)		Total (n = 400)	
	Frequency	Percentage	Frequency	Percentage	Frequency	Percentage
**Serogroup**						
*Varillal*	24	85.7%	316	84.9%	340	85.0%
*Hurstbridge*	0	0.0%	47	12.6%	47	11.8%
*Icterohaemorrhagiae*	2	7.1%	41	11.0%	43	10.8%
*Bratislava*	3	10.7%	28	7.5%	31	7.8%
*Copenhageni*	1	3.6%	12	3.2%	13	3.3%
*Australis*	1	3.6%	7	1.9%	8	2.0%
*Panama*	2	7.1%	5	1.3%	7	1.8%
*Autumnalis*	0	0.0%	5	1.3%	5	1.3%
*Canicola*	0	0.0%	4	1.1%	4	1.0%
*Bataviae*	0	0.0%	3	0.8%	3	0.8%
*Cynopteri*	1	3.6%	2	0.5%	3	0.8%
*Coxi*	0	0.0%	1	0.3%	1	0.3%
*Javanica*	0	0.0%	1	0.3%	1	0.3%
*Hardjo*	0	0.0%	1	0.3%	1	0.3%
*Djasiman*	0	0.0%	1	0.3%	1	0.3%
**Patients who presented one serogroup**	23	82.1%	294	79.0%	317	79.3%
**Patients who presented two serogroups or more**	5	17.9%	78	21.0%	83	20.8%

Paracetamol was the most frequently recorded antipyretic overall, administered in 303 cases (75.8%), including 17 of 28 children under 5 years (60.7%) and 286 of 372 patients aged 5 years or older (76.9%). Among antibiotics, amoxicillin was the most frequently recorded in children under 5 years (20/28, 71.4%), whereas doxycycline predominated in patients aged 5 years or older (222/372, 59.7%). Ciprofloxacin, ceftriaxone, and ampicillin were recorded only among patients aged 5 years or older, whereas erythromycin was recorded in 3 children under 5 years (10.7%) and 14 patients aged 5 years or older (3.8%). Intravenous 0.9% sodium chloride was administered in 3 children under 5 years (10.7%) and 49 patients aged 5 years or older (13.2%) (Table 5).

**Table 5:** Medications recorded among confirmed leptospirosis cases by age group (<5 vs ≥ 5 years) evaluated at primary health care centers in the Peruvian Amazon, 2022–2024.

Medications	<5 years (n = 28)		≥5 years (n = 372)		Total (n = 400)	
	Frequency	Percentage	Frequency	Percentage	Frequency	Percentage
**Antipyretics**						
Paracetamol	17	60.7%	286	76.9%	303	75.8%
Metamizole	4	14.3%	47	12.6%	51	12.8%
**Antibiotics**						
Doxycycline	5	17.9%	222	59.7%	227	56.8%
Amoxicillin	20	71.4%	95	25.5%	115	28.8%
Ciprofloxacin	0	0.0%	35	9.4%	35	8.8%
Erythromycin	3	10.7%	14	3.8%	17	4.3%
Ceftriaxone	0	0.0%	10	2.7%	10	2.5%
Ampicillin	0	0.0%	2	0.5%	2	0.5%
**Intravenous hydration**						
0.9% Sodium chloride	3	10.7%	49	13.2%	52	13.0%
**Other**						
Dimenhydrinate	1	3.6%	14	3.8%	15	3.8%

In exploratory bivariate comparisons by age group, descriptive statistical differences were observed for headache, general malaise and chills (Table 6). Due to the small number of children under 5 years and the exploratory nature of these comparisons, p-values should be interpreted descriptively rather than as confirmatory evidence of age-related differences. Undernutrition was more frequent among children under 5 years (50.0% vs 14.8%; p < 0.001). In addition, presentations more than 3 days after symptom onset were less frequent in children under 5 years than in patients aged 5 years or older (53.6% vs 73.7%; p < 0.001). The complete set of exploratory bivariate comparisons is presented in S2 Table.

**Table 6:** Exploratory bivariate comparisons of selected clinical characteristics between age group (<5 vs ≥5 years) in primary health centers in the Peruvian Amazon, 2022–2024.

Variable	<5 years (n = 28)		≥5 years (n = 372)		p-value
	Frequency	Percentage	Frequency	Percentage	
Headache	10	35.7%	247	66.4%	0.002
General malaise	22	78.6%	190	51.1%	0.005
Chills	2	7.1%	133	35.8%	0.001
Malnutrition	14	50.0%	55	14.8%	<0.001
Duration of illness >3 days	15	53.6%	274	73.7%	<0.001

## Discussion

This study provides descriptive data on the epidemiological and clinical profile of MAT-confirmed leptospirosis in primary health care centers of the Peruvian Amazon, with emphasis on differences between children under 5 years and patients aged 5 years or older. Despite the limited pediatric sample, this study provides rare primary-care data on MAT-confirmed pediatric leptospirosis in a hyperendemic Amazonian setting, where outpatient presentations are underrepresented in the literature. We observed a seasonal clustering of cases that coincided with the climatic cycle of Belén, including periods of river rise and intense rainfall, which likely increase environmental exposure and may contribute to sustained transmission. This context is especially relevant in Belén, where environmental vulnerability and limited sanitation may intensify exposure risks. A clearly defined seasonal pattern was not observed among children under 5 years; however, this likely reflects the small pediatric sample rather than a true absence of temporal variation.

The main age-related differences observed in this primary care cohort involved symptom profile, nutritional classification, and timing of consultation. These comparisons should be viewed as exploratory given the small pediatric sample size, multiple comparisons, and retrospective facility-based data. The small number of children under 5 years limited statistical power for subgroup comparisons and increased the risk of unstable estimates; therefore, age-group comparisons should be interpreted as exploratory and hypothesis-generating rather than confirmatory. In addition, some observed differences may reflect variation in symptom ascertainment, caregiver reporting, and healthcare-seeking behavior rather than true biological differences between age groups. Accordingly, these findings are best interpreted as descriptive and exploratory, particularly for children under 5 years, and require confirmation in larger prospective pediatric cohorts.

Children under 5 years most often presented with nonspecific symptoms, particularly malaise and fever, whereas headache and chills were more frequently recorded in older patients. This pattern is clinically important because it may reduce early diagnostic specificity in first-level care, especially in endemic Amazonian settings where leptospirosis overlaps with other common febrile illnesses such as dengue, malaria, and arboviral infections. In very young children, especially those who are pre-verbal, subjective symptoms are also more difficult to identify and document, which may further contribute to underrecognition. Taken together, these findings support the possibility that mild pediatric leptospirosis may be underdiagnosed in routine outpatient practice unless clinicians maintain a high index of suspicion [10,24,25].

A higher proportion of children under 5 years were evaluated within the first 3 days after symptom onset. This pattern may reflect earlier caregiver concern in young children, although the categorical structure of the available data and the retrospective design limit more detailed interpretation. Caregiver recall, documentation practices, and age-related differences in how symptoms are expressed may also have influenced these findings. Rather than indicating a definitive age-related difference in help-seeking behavior, this observation should be interpreted as a descriptive pattern that warrants further study [8,24,26].

Another notable finding was the higher frequency of undernutrition among children under 5 years. This result should be interpreted carefully because nutritional status was classified using age-specific anthropometric standards, and the retrospective design does not allow causal inference. Moreover, nutritional status in this setting may be shaped by broader social and environmental determinants, including poverty, food insecurity, inadequate sanitation, coinfections, and barriers to care, none of which were captured in the available records [24,27]. Therefore, the observed difference should not be interpreted as evidence that undernutrition independently influenced presentation or recovery, but rather as a contextual finding that may reflect underlying vulnerability in this population.

From a microbiological perspective, Varillal was the predominant MAT serogroup reactivity in both age groups. Panama reactivity appeared proportionally more frequent among children under 5 years, although this observation was based on very few cases and should not be overinterpreted. Since MAT reflects serologic reactivity rather than organism-level characterization, these findings do not permit conclusions about virulence, source of infection, or age-specific susceptibility. Larger studies with broader microbiological characterization are needed to determine whether these patterns are reproducible [28].

Treatment patterns also differed by age group, with amoxicillin more frequently recorded in children under 5 years and doxycycline predominating among older patients [27]. These patterns likely reflect routine primary-care practice and age-related prescribing considerations and are broadly consistent with the Peruvian National Technical Health Standard, which recommends amoxicillin or erythromycin as oral options for mild leptospirosis in children, while doxycycline, amoxicillin, ciprofloxacin, or erythromycin are listed as oral options for adults [21]. However, because this was a retrospective review of primary care records, detailed information on severity, timing of treatment initiation, dosing, duration, adherence, and clinical outcomes was not consistently available. Accordingly, these data should be interpreted as describing local prescribing patterns rather than treatment effectiveness or adherence to guidelines [8,29].

This study reflects a predominantly ambulatory and mild clinical spectrum of leptospirosis managed at primary health care centers. Patients with warning signs or suspected severe disease may have been referred to higher levels of care and are therefore likely underrepresented. In addition, the analysis depended on routinely collected variables and MAT results reported in the medical records, which limited assessment of other potentially important determinants such as socioeconomic conditions, sanitation, coinfections, and complementary diagnostic methods. These considerations are important when interpreting the scope and generalizability of the findings.

Overall, children under 5 years of age with confirmed leptospirosis in this primary care series commonly presented with nonspecific symptoms such as malaise and fever and were frequently identified in flood-prone settings. In the Amazonian context, where leptospirosis coexists with other endemic febrile illnesses, this pattern may complicate early recognition at the primary health care centers. These findings support the need to strengthen clinical suspicion, improve diagnostic capacity in primary health care centers, and reinforce epidemiological surveillance for pediatric leptospirosis in endemic areas. Further studies are needed to better characterize age-related differences in presentation and to clarify how social and environmental factors shape risk in young children.

### Limitations

This study should be interpreted considering several limitations. Its retrospective design, based on routine medical records from primary-care facilities, restricted the completeness and standardization of key variables, including symptom documentation, timing of presentation, and treatment-related information. Symptom ascertainment may also have differed by age group, as young children may have limited ability to report subjective symptoms such as headache, myalgia, chills, dizziness, or abdominal pain, making documentation more dependent on caregiver report and clinician interpretation. The small number of confirmed cases in children under 5 years also limited statistical precision and reduced confidence in subgroup comparisons. Given that the study was conducted in primary health care centers, the findings mainly represent mild and ambulatory cases, whereas more severe presentations may have been referred to higher levels of care and thus were not fully captured. In addition, potentially relevant contextual factors, such as socioeconomic conditions, sanitation, coinfections, and adherence to treatment, were not available in the records reviewed. Although MAT is the reference serological method for leptospirosis surveillance, the use of single-sample MAT results in this retrospective setting may have limited the ability to distinguish recent infection from previous exposure or cross-reactive serological responses, and MAT serogroup reactivity should not be interpreted as definitive identification of the infecting serogroup. Although the total number of suspected cases reviewed during the study period was available, complete year-specific and center-specific denominators for suspected and tested patients were not analyzed in this study, which limits interpretation of temporal and between-center differences in confirmed case counts. Therefore, the study is best understood as a descriptive analysis of outpatient leptospirosis patterns in an endemic Amazonian setting, rather than as evidence of causal age-related differences.

## Supporting information

S1 Table Descriptive distribution of confirmed leptospirosis cases according to health facility, demographic characteristics, year of diagnosis, and reported symptoms in the Loreto region, Peru. (DOCX)

S2 Table Bivariate analysis of sociodemographic, clinical, and microbiological variables by age group in patients with leptospirosis, Loreto, Peru (2022–2024). (DOCX)

S1 File Authorship order change statement. (DOCX)

S2 File Leptospirosis- cover letter. (DOCX)

S3 File Materials license terms. (DOCX)

S4 File Response to reviewers. (DOCX)
