# Supplementary material for: Clinical and epidemiological characteristics of leptospirosis in patients under and over 5 years of age in primary health centers in the Peruvian Amazon, 2022–2024
Source: PLoS Negl Trop Dis. 2026 Jun 25;20(6):e0013473. doi: 10.1371/journal.pntd.0013473 (PMC13421768; doi:10.1371/journal.pntd.0013473)
Supplement: S1 Table — (DOCX) [file pntd.0013473.s001.docx]

**Supplementary Table S1. Descriptive distribution of confirmed leptospirosis cases according to health facility, demographic characteristics, year of diagnosis, and reported symptoms in the Loreto region, Peru.**

|  | | **Health Center** | | | | | | | | | |
| --- | --- | --- | --- | --- | --- | --- | --- | --- | --- | --- | --- |
|  |  | **6 de Octubre** | | **Cardozo** | | **Belén** | | **9 de Octubre** | | Total | |
|  |  | **Frequency** | **Percentage** | **Frequency** | **Percentage** | **Frequency** | **Percentage** | **Frequency** | **Percentage** | **Frequency** | **Percentage** |
| **Confirmed cases n (% of total)** | | 288 | 72.0% | 71 | 17.8% | 4 | 1.0% | 37 | 9.3% | 400 | 100% |
| **Age group** | Under 5 years | 21 | 7.3% | 4 | 5.6% | 1 | 25.0% | 2 | 5,4% | 28 | 7.0% |
|  | 5 years or older | 267 | 92.7% | 67 | 94.4% | 3 | 75.0% | 35 | 94,6% | 372 | 93.0% |
| **Sex** | Male | 126 | 43.8% | 40 | 56.3% | 0 | 0.0% | 17 | 45.9% | 183 | 45.8% |
|  | Female | 162 | 56.3% | 31 | 43.7% | 4 | 100.0% | 20 | 54.1% | 217 | 54.3% |
| **Year** | 2022 | 67 | 23.3% | 0 | 0.0% | 0 | 0.0% | 14 | 37.8% | 81 | 20.3% |
|  | 2023 | 73 | 25.3% | 19 | 26.8% | 0 | 0.0% | 13 | 35.1% | 105 | 26.3% |
|  | 2024 | 148 | 51.4% | 52 | 73.2% | 4 | 100.0% | 10 | 27.0% | 214 | 53.5% |
| **Most frequently reported symptoms** | Fever | 234 | 81.3% | 59 | 83.1% | 4 | 100.0% | 34 | 91.9% | 331 | 82.8% |
|  | Headache | 181 | 62.8% | 42 | 59.2% | 4 | 100.0% | 30 | 81.1% | 257 | 64.3% |
|  | Malaise | 151 | 52.4% | 43 | 60.6% | 4 | 100.0% | 14 | 37.8% | 212 | 53.0% |
|  | Chills | 96 | 33.3% | 22 | 31.0% | 0 | 0.0% | 17 | 45.9% | 135 | 33.8% |
|  | Nausea/Vomiting | 70 | 24.3% | 11 | 15.5% | 3 | 75.0% | 8 | 21.6% | 92 | 23.0% |

Note: Frequencies correspond to the number of observed cases. Percentages in the confirmed cases row were calculated relative to the total number of confirmed cases included in the study (n = 400). For the remaining variables, percentages were calculated using as the denominator the total number of cases corresponding to each health facility.
