# Supplementary material for: Clinical and epidemiological characteristics of leptospirosis in patients under and over 5 years of age in primary health centers in the Peruvian Amazon, 2022–2024
Source: PLoS Negl Trop Dis. 2026 Jun 25;20(6):e0013473. doi: 10.1371/journal.pntd.0013473 (PMC13421768; doi:10.1371/journal.pntd.0013473)
Supplement: S2 Table — (DOCX) [file pntd.0013473.s002.docx]

**Supplementary Table S2. Bivariate analysis of sociodemographic, clinical, and microbiological variables by age group in patients with leptospirosis, Loreto, Peru (2022–2024).**

| **Variable** | **Under 5 years**  **n = 28** | **5 years or older**  **n = 372** | **p-valor*** |
| --- | --- | --- | --- |
| **Year of diagnosis** |  |  | 0.005 |
| 2022 | 0 (0.0%) | 81 (21.8%) |  |
| 2023 | 8 (28.6%) | 97 (26.1%) |  |
| 2024 | 20 (71.4%) | 194 (52.2%) |  |
| **Male sex** | 10 (35.7%) | 173 (46.5%) | 0.327 |
| **District of origin** |  |  | 0.298 |
| Belén | 27 (96.4%) | 361 (97.0%) |  |
| San Juan | 0 (0.0%) | 9 (2.4%) |  |
| Iquitos | 1 (3.6%) | 2 (0.5%) |  |
| **Area of origin** |  |  | 0.129 |
| Urbano | 11 (39.3%) | 163 (43.8%) |  |
| Urbano-marginal | 5 (17.9%) | 114 (30.6%) |  |
| Rural | 12 (42.9%) | 95 (25.5%) |  |
| **Residence in flood-prone area** | 14 (50.0%) | 160 (43.0%) | 0,554 |
| **Symptoms** |  |  |  |
| Fever | 20 (71.4%) | 311 (83.6%) | 0.118 |
| Headache | 10 (35.7%) | 247 (66.4%) | 0.002 |
| Retro-orbital pain | 1 (3.6%) | 28 (7.5%) | 0.709 |
| Malaise | 22 (78.6%) | 190 (51.1%) | 0.005 |
| Anorexia | 3 (10.7%) | 48 (12.9%) | 1.000 |
| Asthenia | 2 (7.1%) | 36 (9.7%) | 1.000 |
| Dizziness | 2 (7.1%) | 84 (22.6%) | 0.058 |
| Chills | 2 (7.1%) | 133 (35.8%) | 0.001 |
| Low back pain | 2 (7.1%) | 60 (16.1%) | 0.282 |
| Musculoskeletal pain | 1 (3.6%) | 38 (10.2%) | 0.503 |
| Calf pain | 0 (0.0%) | 29 (7.8%) | 0.247 |
| Precordial pain | 0 (0.0%) | 5 (1.3%) | 1.000 |
| Myalgia | 4 (14.3%) | 61 (16.4%) | 1.000 |
| Arthralgia | 2 (7.1%) | 37 (9.9%) | 1.000 |
| Rash | 2 (7.1%) | 14 (3.8%) | 0.310 |
| Nausea/vomiting | 6 (21.4%) | 86 (23.1%) | 1.000 |
| Abdominal pain | 3 (10.7%) | 45 (12.1%) | 1.000 |
| Constipation | 1 (3.6%) | 6 (1.6%) | 0.401 |
| Petechiae | 0 (0.0%) | 4 (1.1%) | 1.000 |
| Epistaxis | 0 (0.0%) | 2 (0.5%) | 1.000 |
| Hematuria | 0 (0.0%) | 2 (0.5%) | 1.000 |
| Oliguria | 1 (3.6%) | 3 (0.8%) | 0.253 |
| Gastrointestinal hemorrhage | 0 (0.0%) | 1 (0.3%) | 1.000 |
| Cough | 6 (21.4%) | 47 (12.6%) | 0.240 |
| Dyspnea | 0 (0.0%) | 14 (3.8%) | 0.612 |
| Diarrhea | 4 (14,3%) | 44 (11.8%) | 0.761 |
| Blood pressure |  |  | 0.844 |
| Hypotension | 1 (3.6%) | 15 (4.0%) |  |
| Normotension | 24 (85.7%) | 296 (79.6%) |  |
| Hypertension | 3 (10.7%) | 61 (16.4%) |  |
| Heart rate |  |  | 0.476 |
| Bradycardia | 0 (0.0%) | 10 (2.7%) |  |
| Normal heart rate | 28 (100%) | 341 (91.7%) |  |
| Tachycardia | 0 (0.0%) | 21 (5.6%) |  |
| Malnutrition | 14 (50.0%) | 55 (14.8%) | <0.001 |
| **Duration of illness greater than 3 days** | 11 (39.3%) | 274 (73.7%) | <0.001 |
| **Identified serovar** |  |  |  |
| *Varillal* | 24 (85.7%) | 316 (84.9%) | 1.000 |
| *Icterohemorraghiae* | 2 (7.1%) | 41 (11.0%) | 0.754 |
| *Bataviae* | 0 (0.0%) | 3 (0.8%) | 1.000 |
| *Bratislava* | 3 (10.7%) | 28 (7.5%) | 0.468 |
| *Autumnalis* | 0 (0.0%) | 5 (1.3%) | 1.000 |
| *Hurstbridge* | 0 (0.0%) | 47 (12.6%) | 0.060 |
| *Panama* | 2 (7.1%) | 5 (1.3%) | 0.080 |
| *Canicola* | 0 (0.0%) | 4 (1.1%) | 1.000 |
| *Coxi* | 0 (0.0%) | 1 (0.3%) | 1.000 |
| *Australis* | 1 (3.6%) | 7 (1.9%) | 0.443 |
| *Javanica* | 0 (0.0%) | 1 (0.3%) | 1.000 |
| *Cynopteri* | 1 (3.6%) | 2 (0.5%) | 0.196 |
| *Hardjo* | 0 (0.0%) | 1 (0.3%) | 1.000 |
| *Djasiman* | 0 (0.0%) | 1 (0.3%) | 1.000 |
| *Copenhag* | 1 (3.6%) | 12 (3.2%) | 1.000 |

Notes:

- Data are presented as n (%).
- Percentages were calculated by column according to the total number in each age group.
- The p value was calculated using Fisher’s exact test to compare proportions between the <5 years and ≥5 years groups.
- Variables with multiple categories are presented in separate rows, and the p value corresponds to the overall comparison of the variable between groups.
- n: number of patients.
