# Supplementary material for: Clinical and epidemiological characteristics of leptospirosis in patients under and over 5 years of age in primary health centers in the Peruvian Amazon, 2022–2024
Source: PLoS Negl Trop Dis. 2026 Jun 25;20(6):e0013473. doi: 10.1371/journal.pntd.0013473 (PMC13421768; doi:10.1371/journal.pntd.0013473)
Supplement: S1 File — (DOCX) [file pntd.0013473.s003.docx]

**WRITTEN STATEMENT OF AUTHORSHIP ORDER CHANGE**

*Manuscript title: “Differential profile of Leptospirosis in patients under and over 5 years of age in health centers of the Peruvian Amazon, 2022–2024”*

**Manuscript ID: PNTD-D-25-01422R1**

We, the undersigned co-authors of the manuscript referenced above, hereby confirm that we all agree to the change made to the author byline.

No authors have been added or removed. The only modification is the order of the authors in the byline, including the designation of the corresponding author and the affiliation numbering shown in the manuscript files.

All authors confirm that they meet the authorship criteria for this manuscript, have reviewed the revised author order, and approve the submission of the revised manuscript with the author list shown below.

**Author byline**

| **Previous order** | **Current order** |
| --- | --- |
| 1. Stefano V. Davila-Philipps¹,³  2. Kary K. Vela-Tello¹,³  3. Jorge I. Carrasco-Celi¹,³  4. Yoki N. Rios-Alava¹,³  5. James L. Vasquez-Lechuga¹,³  6. Marcos H. Parimango-Alvarez¹,²  7. Edgar A. Ramirez-García¹,²  8. Johan Marin-Lizarraga¹,²  9. Tery Vasquez-Hassinger²*  10. Karine Zevallos¹ | 1. Stefano V. Davila-Philipps¹  2. Tery Vasquez-Hassinger²,³  3. Kary K. Vela-Tello¹  4. Jorge I. Carrasco-Celi¹  5. Yoki N. Rios-Alava¹  6. James L. Vasquez-Lechuga¹  7. Marcos H. Parimango-Alvarez¹,²  8. Johan Marin-Lizarraga¹,²  9. Edgar A. Ramirez-García¹,²  10. Karine Zevallos¹* |

**Signatures**

Each author should sign and date below to indicate agreement with this authorship order change.

| **Author name** | **Signature** | **Date** |
| --- | --- | --- |
| Stefano V. Davila-Philipps | 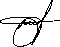 | April 13 2026 |
| Tery Vasquez-Hassinger | 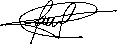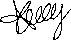 | April 13 2026 |
| Kary K. Vela-Tello |  | April 13 2026 |
| Jorge I. Carrasco-Celi | 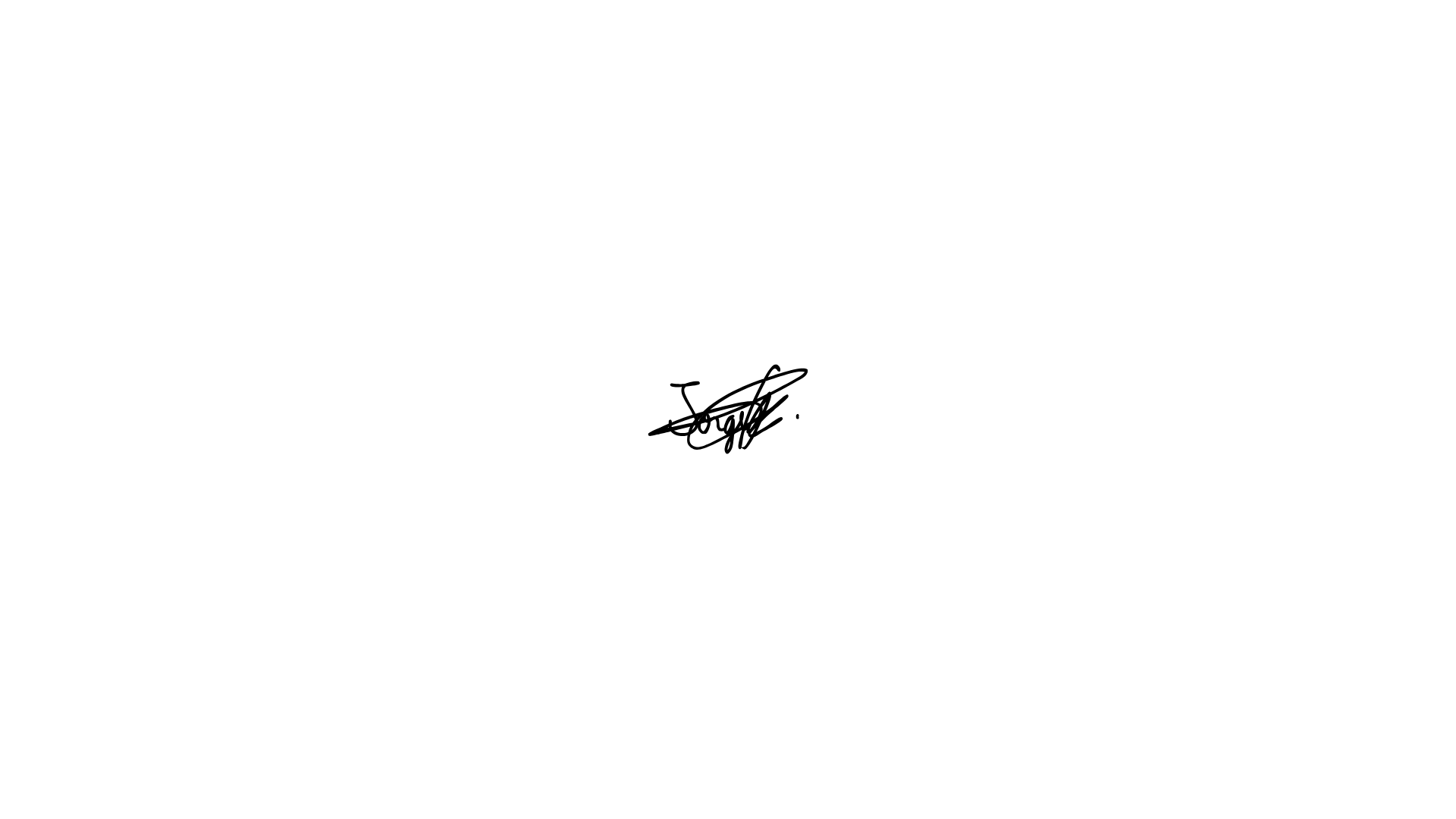 | April 13 2026 |
| Yoki N. Rios-Alava | 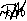 | April 13 2026 |
| James L. Vasquez-Lechuga | 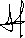 | April 13 2026 |
| Marcos H. Parimango-Alvarez | 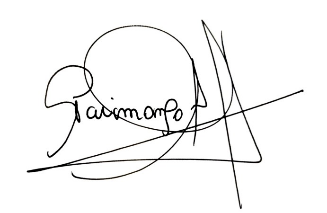 | April 16 2026 |
| Johan Marin-Lizarraga | 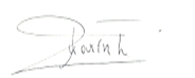 | April 14 2026 |
| Edgar A. Ramirez-García | 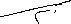 | April 13 2026 |
| Karine Zevallos | 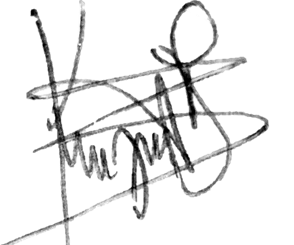 | April 13 2026 |

**Corresponding author contact for this request:** Karine Zevallos
