# Supplementary material for: Clinical and epidemiological characteristics of leptospirosis in patients under and over 5 years of age in primary health centers in the Peruvian Amazon, 2022–2024
Source: PLoS Negl Trop Dis. 2026 Jun 25;20(6):e0013473. doi: 10.1371/journal.pntd.0013473 (PMC13421768; doi:10.1371/journal.pntd.0013473)
Supplement: S2 File — (DOCX) [file pntd.0013473.s004.docx]

August 8,2025

Dear editors of PLOS Neglected Tropical Diseases,

We are excited to submit our manuscript titled “Differential profile of Leptospirosis in patients under and over 5 years of age in health centers of the Peruvian Amazon, 2023–2024” for your consideration.

This work comes from our experience in the Peruvian Amazon, where leptospirosis is a serious public health issue, especially during the rainy season. While leptospirosis can affect people of all ages, we noticed that very little is known about how the disease presents in children under five. Our study was born out of a need to understand this gap. By analyzing 400 confirmed cases, we were able to shed light and highlight key differences on age-related differences in clinical presentation, serovar distribution, and nutritional status between young children and older patients.

We believe this study will resonate with the NTDs community because it brings attention to an age group that is often overlooked in both research and public health strategies. Our findings suggest that leptospirosis behaves differently in young children, often with vague symptoms and less typical signs, making it harder to detect early. By recognizing these differences, we hope to encourage more targeted approaches to diagnosis, treatment, and prevention, especially in primary care settings in endemic regions.

By contributing to the understanding of atypical clinical presentations in endemic settings, this study can support the development of age-specific public health interventions and diagnostic guidelines, ultimately enhancing the prevention and control of leptospirosis in low-resource regions like the Amazon.

Thank you for your time and consideration. We look forward to the opportunity to contribute to the ongoing dialogue on improving health outcomes in neglected tropical disease contexts.

Sincerely,
Tery Vasquez Hassinger (Corresponding Author)

Email: [teryv4@gmail.com](mailto:teryv4@gmail.com)
