# Supplementary material for: Clinical and epidemiological characteristics of leptospirosis in patients under and over 5 years of age in primary health centers in the Peruvian Amazon, 2022–2024
Source: PLoS Negl Trop Dis. 2026 Jun 25;20(6):e0013473. doi: 10.1371/journal.pntd.0013473 (PMC13421768; doi:10.1371/journal.pntd.0013473)
Supplement: S3 File — (DOCX) [file pntd.0013473.s005.docx]

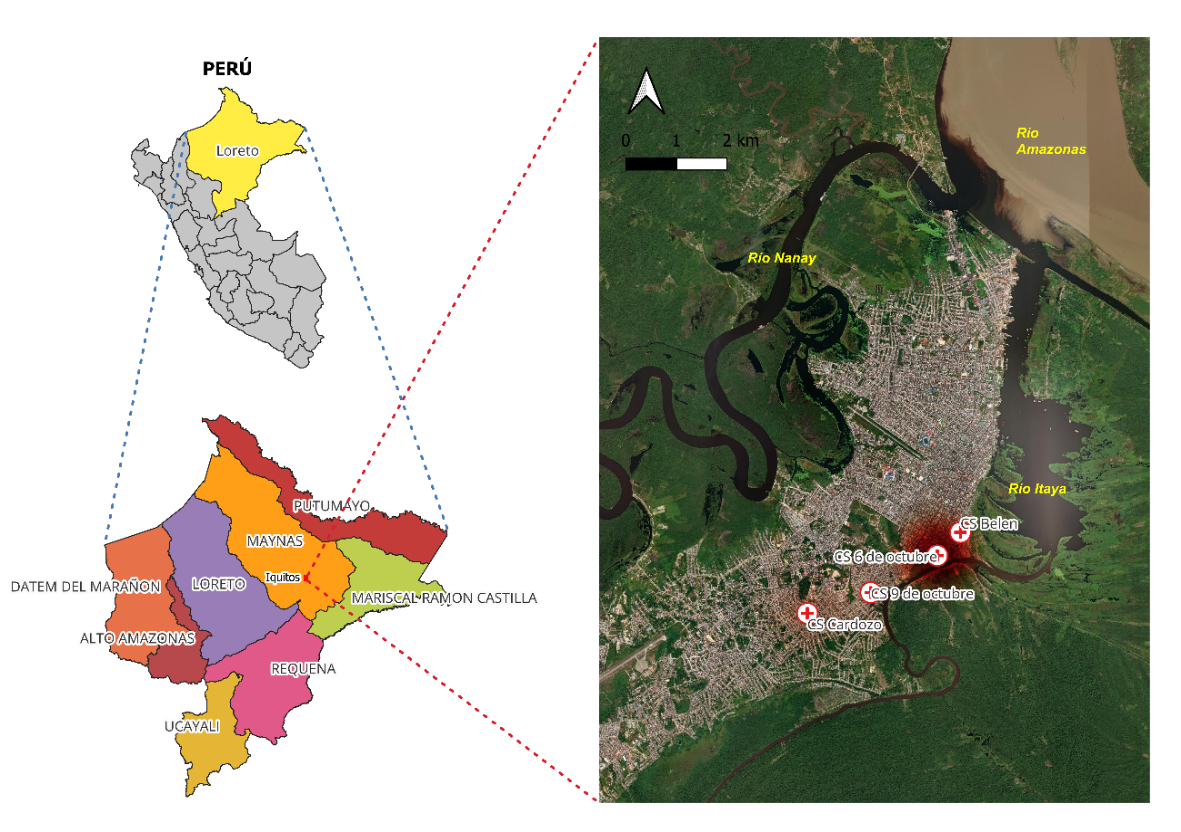


Figure 1. Geographic location of the study area and included primary health centers in Loreto, Peru. Map boundaries and geographical data were obtained from GeoGPS Perú (public domain). The base map imagery was sourced from Esri World Imagery, which permits use in academic publications (Terms of use: <https://doc.arcgis.com/en/arcgis-online/reference/static-maps.htm>). All sources used are open access and compatible with the CC BY 4.0 license requirements


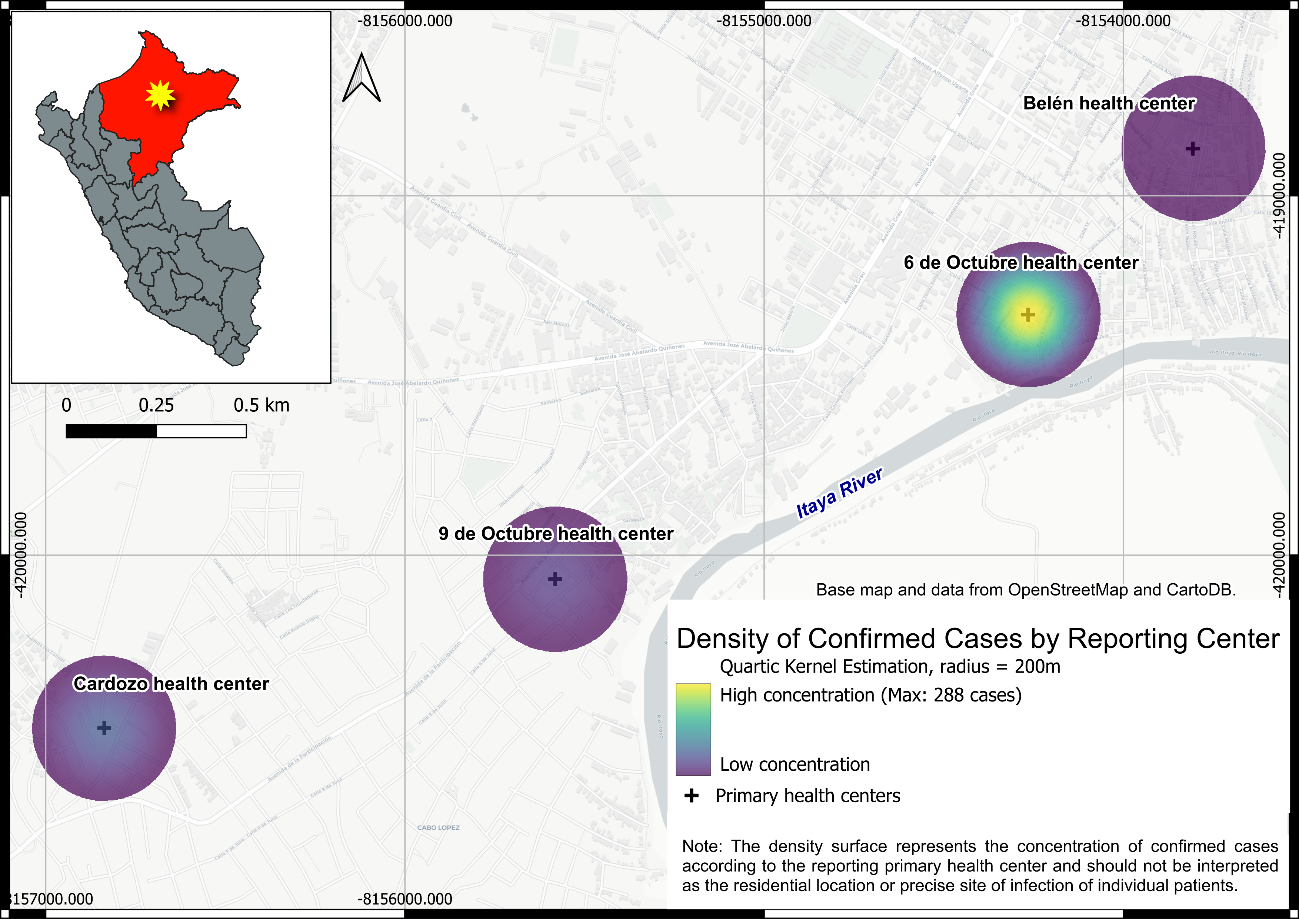


Figure 3. Kernel density map of MAT-confirmed leptospirosis cases by reporting primary health center in Belén, Loreto, Peruvian Amazon, 2022–2024. Map boundaries and geographical data were obtained from GeoGPS Perú (public domain). Base map and street data from OpenStreetMap and CartoDB. OpenStreetMap data is licensed under the Open Data Commons Open Database License (ODbL) by the OpenStreetMap Foundation (Terms of use: <https://www.openstreetmap.org/copyright>). All materials are open access and compatible with the CC BY 4.0 license.
