# Supplementary material for: Clinical and epidemiological characteristics of leptospirosis in patients under and over 5 years of age in primary health centers in the Peruvian Amazon, 2022–2024
Source: PLoS Negl Trop Dis. 2026 Jun 25;20(6):e0013473. doi: 10.1371/journal.pntd.0013473 (PMC13421768; doi:10.1371/journal.pntd.0013473)
Supplement: Attachment — Submitted filename: My Comments_PNTD-D-25-01422.pdf [file pntd.0013473.s007.pdf]

The authors present a retrospective, primary care-based study describing the clinical and epidemiological characteristics of MAT-confirmed leptospirosis cases in the district of Belén, Loreto, with a particular focus on comparisons between patients under and over five years of age. The manuscript addresses an important and underexplored population in a hyperendemic Amazonian setting and provides valuable descriptive data derived from routine health services, which are rarely reported in the literature. Strengths of the study include the use of laboratory-confirmed cases, the multi-year observation period, and the emphasis on outpatient presentations in vulnerable communities, all of which enhance the relevance of the work for public health practice in endemic regions. The has the potential to contribute meaningful insights into age-related differences in leptospirosis at the primary care level. However, I have several important methodological and interpretative concerns that need to be carefully addressed and revised before the manuscript can be considered for acceptance. I strongly suggest that the next version include numbered lines to facilitate the review process.

Major Comments

1) The most critical methodological concern relates to the use of binary logistic regression with “being under 5 years of age” as the outcome variable, based on only 28 children under five. Modeling age group as a dependent variable is conceptually problematic, as age is not a biological or clinical outcome but an inherent characteristic. As a result, several reported associations (e.g., duration of illness increasing the likelihood of belonging to the <5 group) lack biological plausibility and may reflect statistical artifacts rather than meaningful epidemiological relationships.

   Moreover, the very small number of events (<5 years) substantially limits the reliability of multivariate modeling. Even with only two predictors retained, the analysis is highly susceptible to overfitting, unstable odds ratios, and inflated effect sizes. The extremely strong association observed for underweight status (OR = 0.051) further suggests model instability and/or issues with variable coding and reference categories.

   The authors should reconsider the use of multivariate regression altogether. At minimum, the regression model should be removed or reframed, and the study should be clearly positioned as descriptive and exploratory, with cautious interpretation of bivariate associations only.

2) The Methods section states that all variables, including duration of illness, were treated as categorical. However, in the multivariate model, duration of illness is clearly treated as a continuous quantitative variable, with interpretation “for each additional day of illness.”

   This inconsistency undermines the transparency and reproducibility of the analysis. Furthermore, duration of illness was not significant in the bivariate analysis (p = 0.32) but became highly significant in the multivariate model, which is not adequately explained and raises concerns about analytical coherence.

   The authors must clarify how duration of illness was coded and analyzed, ensure consistency between methods and results, and justify the biological and epidemiological meaning of this association. Without clarification, this result should not be retained.

3) Nutritional status is treated using BMI categories (underweight, normal, overweight) in children under five. This is problematic, as BMI is not the recommended anthropometric indicator for this age group. International standards (e.g., WHO) recommend weight-for-height, weight-for-age, or height-for-age Z-scores.

   The use of BMI without justification may introduce classification bias and weakens the validity of conclusions regarding malnutrition and leptospirosis in young children.

   The authors should justify the use of BMI in children under five or, preferably, reanalyze nutritional status using age-appropriate WHO growth indicators.

4) The manuscript reports statistically significant associations between age group and specific "species/serovars" (Panama and Hurstbridge). However:
   a) The absolute numbers are extremely small (e.g., 2 cases of Panama in children <5).
   b) Pearson's chi-square test is used despite small cell counts, where Fisher's exact test would be more appropriate.
   c) Multiple serovars and symptoms are tested without adjustment for multiple comparisons, increasing the risk of false-positive findings.
   d) MAT-based serovar attribution is inherently limited by cross-reactivity, which the authors acknowledge but still interpret as evidence of "acute infection caused by a specific serovar."

   Serovar-specific conclusions should be clearly framed as exploratory and hypothesis-generating only. Strong statements regarding virulence, transmission patterns, or vaccine relevance are not supported by the data and should be substantially softened.

5) Several symptoms compared between <5 and ≥5 years (e.g., headache, myalgia, musculoskeletal pain) depend on subjective reporting, which is inherently limited in young children. Differences observed may reflect reporting and ascertainment bias rather than true biological differences.

   This limitation is not sufficiently discussed and undermines the interpretation of age-specific clinical profiles. The Discussion should explicitly acknowledge differential symptom reporting capacity by age and avoid implying pathophysiological differences based solely on these comparisons.

6) All cases were mild, with no hospitalizations or deaths, as expected from a primary care-based study. However, many conclusions are written broadly, implying relevance to leptospirosis in general rather than outpatient, mild disease only.

   The scope of inference should be more clearly restricted throughout the Abstract, Discussion, and Conclusions to mild leptospirosis managed at the primary care level.

Minor Comments

Title and Abstract

Page 1, Title: The title refers to “2023–2024,” while the Methods and Results include 2022–2024. This inconsistency should be corrected.

Page 2, Abstract: The phrase “each additional day of illness was associated with an increased likelihood of belonging to this age group” is conceptually unclear and should be rephrased or removed.

The abstract should be adapted according to all the suggestions made throughout the text.

Introduction

The introduction is too dense and descriptive in places and could be more argument-driven: it lists facts but does not sufficiently explain why under-5s deserve a dedicated analysis beyond “less studied.”

The transition from Peru burden to age differences is a bit abrupt; you could add one bridging sentence explaining why the high-burden setting makes age-stratified outpatient profiles clinically important.

Page 4: The phrase “Among the most affected species, rodents are considered chronic carriers…” This is conceptually confusing: rodents are not “most affected species,” they are key reservoir hosts. On the other hand, dogs, pigs, and cattle can both be carriers and suffer the consequences of the infection. A more appropriate reference should be included here.

Page 4 -5: The section on pediatric vs adult differences is broadly accurate, but it risks sounding definitive. “Some studies suggest…” is good. However, phrases like “may vary significantly” could be supported by specifying which outcomes differ (e.g., jaundice, renal failure, mortality in hospitalized series). Add a sentence about outpatient vs hospitalized populations since your data are primary care.

Page 5: “limited number of studies that systematically compare clinical and epidemiological differences…” This is correct but could be more compelling if you specify: under-5 subgroup; primary care/outpatient context; Amazonian hyperendemic setting,; and diagnostic/triage relevance.

Page 5, final paragraph: The objective is described as “to describe,” which matches the retrospective design. But “strengthen strategies at three levels” is aspirational and may read as too broad

The objective is descriptive, yet later analyses imply causal inference. Consider aligning wording more clearly with a descriptive/exploratory design.

Methods

Page 5, Study design: The term “multicenter” should be contextualized, as one health center accounts for 72% of cases. Potential center-level bias should be acknowledged.

Page 5: Given the strong emphasis on environmental exposure, flooding, and spatial vulnerability in the district of Belén, the inclusion of a study-area map would substantially strengthen the manuscript. A map showing the location of Belén within Loreto, the main rivers, and the four participating health centers would improve clarity and help contextualize the epidemiological findings, particularly the unequal contribution of cases across centers.

Page 6, **Case definition**: Clarify the rationale for using a single MAT titer ≥1:800 without paired samples, particularly in a hyperendemic area.

Page 6: In the sentence: "These conditions favor the proliferation of vectors and the transmission of infectious diseases…" Leptospirosis is not vector-borne; transmission is environmental exposure to contaminated urine/water/soil. "Vectors" could mislead readers.

Page 6, Statistical analysis:

Clarify why Pearson's chi-square was used instead of Fisher's exact test for sparse data.

Explicitly state how missing data were handled.

Clarify reference categories used in the logistic regression.

Results

Page 7: The Results describe a "progressive increase" and a 103.8% rise in 2024 vs 2023. However, the Results do not provide denominators or context (e.g., number tested, changes in testing practices, reporting completeness, outbreak alerts, flooding intensity, or changes in referral pathways). In a retrospective record review, increases may reflect surveillance intensity rather than true incidence. Add supporting context or report: number of suspected cases tested per year, positivity rate per year, any changes in testing/referral policies, or explicitly state this is "increase in confirmed cases identified in records," not necessarily incidence.

Avoid causal language. For example: Replace "a progressive increase was observed" with "a higher number of confirmed cases was recorded in 2024 compared with prior years."

Page 7: One health center accounts for 72% of cases (C.S 06 de Octubre), while another contributes 1%. This makes pooled estimates heavily dependent on a single site. Without describing catchment, access, or testing behavior, this may reflect center-level ascertainment rather than epidemiology. Report per-center characteristics (even briefly), and consider adding a sensitivity description ("results were similar when restricting to the largest center"), or stratified descriptive tables by center (at least as supplementary material).

Page 7 and 9: The authors describe "monthly prevalence" and "marked seasonal pattern," but the data presented are case counts, not prevalence (no population denominator). The figure caption uses "Monthly prevalence," which is incorrect. Replace "prevalence" with "monthly number of confirmed cases" (or "monthly distribution of confirmed cases").

The statement that <5 cases were "scarce and dispersed" may simply reflect small n (28 total), limiting inference about seasonality. It should be reframe as descriptive.

Page 9 (Figure 2): The inclusion of a map is appropriate and adds value to the study; however, the current figure does not meet scientific publication standards. It appears to be based on a Google Maps screenshot with overlaid heat-style markers, which limits reproducibility and

cartographic clarity. I recommend replacing it with a GIS-generated map showing the study area and the four health centers, using standardized symbology (e.g., proportional symbols), administrative boundaries, and basic cartographic elements (scale bar, north arrow, legend). This would substantially improve the scientific quality of the figure.

Page 10: The text says "average time … was between 3 to 6 days," but Table 2 reports categories (0–3, 3–6, >6). That is not an "average." Use: "The most frequent category of time from symptom onset to consultation was 3–6 days (45.8%)."

Page 10: Several comparisons (rural vs urban; flood-prone residence; sex distribution) are described, but it is unclear which differences were statistically tested (Table 6 lists only selected chi-square results). Either: report p-values for these key contextual variables, or explicitly state that they are descriptive and not statistically compared (and why). Also, clarify operational definitions: How was "flood-prone area" determined (self-report, address, municipal classification)?

Page 10 and 11, paragraph on "insidious onset and progressive course": Reporting 100% of cases with identical onset and course suggests limited discriminatory value or default recording; this should be explained or removed.

Page 11: Table 2 shows 100% insidious onset and 100% progressive course, with no variability. This is implausible clinically and suggests default charting, poor variable definition, or that these fields were not meaningfully captured. Either remove these variables from Results, or explain why they are uniformly recorded. At minimum, acknowledge in Discussion as potential information bias.

Page 11, Table 1: Percentages are sometimes reported without corresponding denominators; consider adding absolute numbers consistently.

Page 11: The Results state that symptoms like headache, chills, myalgia are more frequent in ≥5, while malaise and fever are frequent in <5. But many symptoms (headache, myalgia) are harder to ascertain in very young children. This is a major interpretation caveat and should be signposted already in Results or at least carefully in Discussion.

Page 13: Table 4 labels "Species," but entries are serovars/serogroups (e.g., Icterohemorraghiae, Canicola, Autumnalis, Panama). Calling these "species" is incorrect and will undermine credibility. Please use "serovar" (or "serogroup/serovar panel reactivity by MAT") consistently in all text and tables.

Page 13: The antibiotic patterns are reported, but the Results do not indicate: clinical severity markers, criteria guiding antibiotic choice, whether prescriptions align with national guidelines, dosing, duration, or timing relative to symptom onset. Keep descriptive, but consider adding guideline citation for pediatric management, note that treatment reflects routine practice and age-related contraindications (e.g., doxycycline in younger children).

Page 14-15: Table 6 reports chi-square significance for Panama and Hurstbridge. However, the absolute numbers are tiny (Panama in <5: 2 cases; Panama in ≥5: 4 cases). With such sparse counts, chi-square assumptions may be violated; Fisher's exact test is more appropriate. Re-run these comparisons using Fisher's exact test and report exact p-values. Additionally, consider flagging these serovar comparisons as exploratory.

Page 14-15: Many symptoms/variables are presented in Table 3 and Table 4, but Table 6 lists only a subset of tests and does not address the risk of false positives from multiple testing. Either apply an FDR approach (e.g., Benjamini–Hochberg) or explicitly state analyses are exploratory and interpret p-values cautiously.

Page 15: The regression models “being <5 years” as an outcome. This is conceptually questionable (age is not a clinical outcome). The text says underweight is associated with a “higher probability” of being under 5, but the OR is 0.051, suggesting either outcome coding is reversed (≥5 coded as 1), or interpretation is flawed. The authors then attempt to explain it by describing “reduction in probability of belonging to older group,” which is confusing and non-standard. Please explicitly state outcome coding (which group = 1), state reference categories, and rewrite interpretation clearly (or remove the model).

Bivariate duration is categorical and non-significant, but in regression duration is “quantitative” and significant. This is an important methodological inconsistency that must be corrected.

Discussion

Page 16, Paragraph 1: Overstatement of “defined seasonal pattern.” The Results show monthly peaks in the ≥5 group, but you should avoid language implying robust seasonality without denominators and without controlling for surveillance intensity or testing volume. A safer formulation would be: “a seasonal clustering of cases was observed…” rather than “defined seasonal pattern.”

Causal language (“reinforces the role of the environment as a determining factor”). This is a retrospective, facility-based dataset. The Discussion should not imply causality. Suggest: “is consistent with an environmentally mediated transmission pattern”.

Interpretation for <5 group is speculative and not strongly supported. The statement that lack of seasonality in <5 could be due to reduced exposure/mobility is plausible but may simply reflect low case counts (n=28), limiting the power to detect any seasonal pattern. This limitation should be explicitly stated here.

Missing acknowledgment of ascertainment bias. Children may be taken to care differently; testing thresholds may differ; and symptom-reporting differs. These should be acknowledged when interpreting “seasonality differences.”

Page 16, Paragraph 2: This paragraph imports statistical conclusions too strongly. “Identified as significant findings” reads as confirmatory, but the analysis has major limitations (small n, multiple comparisons, questionable regression outcome).

Internal inconsistency about duration of illness. In the Results, duration categories were not significant in bivariate analysis, yet the Discussion treats “0–3 days” as a key significant finding; this is not well supported.

The regression is conceptually problematic. Modeling “being <5 years” as an outcome should not be treated as evidence of “factors” associated with pediatric leptospirosis. This paragraph should avoid language implying independent predictors.

BMI measure in <5 is questionable. As discussed, BMI is not ideal for children under five without WHO-standard z-scores. This must be acknowledged before drawing conclusions about malnutrition and vulnerability. Reframe as descriptive/hypothesis-generating

Page 17, Paragraph 3: "Most relevant finding" is a value judgment and should be backed by a clinical rationale or softened.

Symptom comparability issue is missing. Differences in headache/myalgia/chills may reflect children's inability to report symptoms and clinicians' recording practices, not true biological differences.

"More classic symptoms…predominated" is not a fair comparison unless you explicitly acknowledge reporting bias.

The citations are fine, but the paragraph needs one explicit sentence acknowledging age-related symptom ascertainment limitations.

Page 17, Paragraph 4: This paragraph is generally reasonable, but it still risks overgeneralization. The study includes only MAT-confirmed mild outpatient cases, so "clinical profile of children affected" should be narrowed to "in this primary care-based series".

"May contribute to underreporting" is plausible but speculative; if retained, it should be clearly framed as a hypothesis.

Good applied recommendation (training), but make sure you avoid implying that training alone solves underdiagnosis; diagnostic access matters too.

Page 17, Paragraph 5: Methodological weakness is not acknowledged: BMI is not an optimal metric for <5; you should either justify its use or state it as a limitation before interpreting. Without that, the paragraph appears overconfident.

Causal speculation ("malnutrition compromises immune response and could influence the less florid presentation"). This is biologically plausible but not demonstrated by this dataset. It also risks circular reasoning and overreach.

Consider confounding: underweight is strongly correlated with poverty, sanitation, co-infections, none measured. The Discussion must acknowledge unmeasured confounding.

Page 17, Paragraph 6: The descriptive observation (46% within 0–3 days) is fine, but the paragraph over-links it to clinical presentation differences in a way that reads causal. Also, Results/Methods have inconsistencies in how "duration" is analyzed (categorical vs continuous). The Discussion should not lean heavily on duration until the analysis is fixed. Keep as descriptive and explicitly mention potential bias.

Page 17, Paragraph 7: This is one of the paragraphs that most needs correction.

The Results/Table label these as "species," but they are MAT serovars/serogroups. The Discussion should correct and use consistent terms.

"Panama serovar significantly more frequent (7% vs 1%)" corresponds to 2 vs 4 cases. That is extremely fragile and likely violates chi-square assumptions (Fisher would be appropriate). This must be explicitly acknowledged.

Claims about "virulence, transmission patterns, immunological susceptibility" are not supported by MAT data and tiny counts.

Page 18, Paragraph 8: The descriptive reporting is fine, but do not imply appropriateness without evidence. You do not assess outcomes, adherence, dosing, timing, or guideline concordance systematically. Some antibiotic differences are expected due to age-related contraindications (e.g., doxycycline). This should be stated explicitly. "Crucial to prevent complications" is true generally, but your dataset contains only mild cases and no outcomes. This sentence is a bit disconnected from the evidence.

Page 18, Paragraph 9: Consider strengthening it by explicitly stating: selection context: primary care only, referral mechanism, and how this affects generalizability to severe disease. Also add: lack of lab confirmation beyond MAT, and missingness concerns (if applicable).

Page 18, Paragraph 10: This paragraph over-summarizes in a way that implies a stable "profile," but many listed elements are not strongly supported:

- "predominance of female sex" with n=28 is weak and likely random.
- "rural or peri-urban origin" is descriptive but not tested as significant.
- "residence in flood-prone areas" differs only modestly (50% vs 43%).
- "low BMI" is methodologically questionable in <5.
- "Panama followed by Varillal" is based on tiny counts and MAT limitations.

The concluding policy recommendation ("justify the need to strengthen surveillance…") is reasonable, but it should be framed as supported by the high-burden context and diagnostic challenges, not by fragile subgroup associations.

Limitations Paragraph

The current Limitations section is framed too narrowly and does not adequately reflect the full range of methodological and analytical constraints of the study. At present, the paragraph focuses almost exclusively on the absence of severe cases and deaths, which is an important point but represents only one dimension of the study's limitations. Several additional factors that directly affect the interpretation and robustness of the findings are not acknowledged and should be explicitly discussed. In particular, the very small number of children under five years of age substantially limits statistical power for age-stratified comparisons, multivariate analyses, and serovar-specific associations, and this limitation should be clearly stated. The retrospective design based on medical record review introduces potential selection bias, as inclusion depended on the completeness of clinical records, as well as information bias related to variable documentation quality across patients, age groups, and health centers. Differential symptom ascertainment by age is another critical limitation that is not addressed; several symptoms compared between age groups rely on subjective reporting and may be under-recognized or under-recorded in young children, which could partly explain observed differences rather than true biological variation. In addition, diagnosis and serovar attribution relied exclusively on MAT performed on single serum samples, without paired sera, PCR, or culture confirmation; given the known cross-reactivity and limited resolution of MAT in endemic settings, this limitation is particularly relevant in light of the serovar-specific interpretations presented. The assessment of nutritional status in children under five using BMI should also be recognized as a limitation, as BMI is not the optimal anthropometric indicator for this age group and may lead to misclassification. Furthermore, the markedly unequal contribution of cases across participating health centers may reflect differences in healthcare access, testing practices, or catchment populations rather than true epidemiological variation, and this potential center-level bias

should be acknowledged. Finally, the limitations section should clarify that multiple comparisons were performed in an exploratory context and that the findings are best interpreted as descriptive and hypothesis-generating rather than confirmatory.

Language and Style

Minor grammatical issues occur throughout (e.g., inconsistent pluralization, occasional awkward phrasing). A careful language revision by a fluent English speaker is recommended.

Consistency should be ensured for terms such as “under 5 years,” “under five,” and “<5 years.”
