# Supplementary material for: Clinical and epidemiological characteristics of leptospirosis in patients under and over 5 years of age in primary health centers in the Peruvian Amazon, 2022–2024
Source: PLoS Negl Trop Dis. 2026 Jun 25;20(6):e0013473. doi: 10.1371/journal.pntd.0013473 (PMC13421768; doi:10.1371/journal.pntd.0013473)
Supplement: S4 File — (DOCX) [file pntd.0013473.s006.docx]

**Supplementary Materials**

**Supplementary Table S1. Descriptive distribution of confirmed leptospirosis cases according to health facility, demographic characteristics, year of diagnosis, and reported symptoms in the Loreto region, Peru.**

|  | | **Health Center** | | | | | | | | | |
| --- | --- | --- | --- | --- | --- | --- | --- | --- | --- | --- | --- |
|  |  | **6 de Octubre** | | **Cardozo** | | **Belén** | | **9 de Octubre** | | Total | |
|  |  | **Frequency** | **Percentage** | **Frequency** | **Percentage** | **Frequency** | **Percentage** | **Frequency** | **Percentage** | **Frequency** | **Percentage** |
| **Confirmed cases n (% of total)** | | 288 | 72.0% | 71 | 17.8% | 4 | 1.0% | 37 | 9.3% | 400 | 100% |
| **Age group** | Under 5 years | 21 | 7.3% | 4 | 5.6% | 1 | 25.0% | 2 | 5,4% | 28 | 7.0% |
|  | 5 years or older | 267 | 92.7% | 67 | 94.4% | 3 | 75.0% | 35 | 94,6% | 372 | 93.0% |
| **Sex** | Male | 126 | 43.8% | 40 | 56.3% | 0 | 0.0% | 17 | 45.9% | 183 | 45.8% |
|  | Female | 162 | 56.3% | 31 | 43.7% | 4 | 100.0% | 20 | 54.1% | 217 | 54.3% |
| **Year** | 2022 | 67 | 23.3% | 0 | 0.0% | 0 | 0.0% | 14 | 37.8% | 81 | 20.3% |
|  | 2023 | 73 | 25.3% | 19 | 26.8% | 0 | 0.0% | 13 | 35.1% | 105 | 26.3% |
|  | 2024 | 148 | 51.4% | 52 | 73.2% | 4 | 100.0% | 10 | 27.0% | 214 | 53.5% |
| **Most frequently reported symptoms** | Fever | 234 | 81.3% | 59 | 83.1% | 4 | 100.0% | 34 | 91.9% | 331 | 82.8% |
|  | Headache | 181 | 62.8% | 42 | 59.2% | 4 | 100.0% | 30 | 81.1% | 257 | 64.3% |
|  | Malaise | 151 | 52.4% | 43 | 60.6% | 4 | 100.0% | 14 | 37.8% | 212 | 53.0% |
|  | Chills | 96 | 33.3% | 22 | 31.0% | 0 | 0.0% | 17 | 45.9% | 135 | 33.8% |
|  | Nausea/Vomiting | 70 | 24.3% | 11 | 15.5% | 3 | 75.0% | 8 | 21.6% | 92 | 23.0% |

Note: Frequencies correspond to the number of observed cases. Percentages in the confirmed cases row were calculated relative to the total number of confirmed cases included in the study (n = 400). For the remaining variables, percentages were calculated using as the denominator the total number of cases corresponding to each health facility.

**Supplementary Table S2. Bivariate analysis of sociodemographic, clinical, and microbiological variables by age group in patients with leptospirosis, Loreto, Peru (2022–2024).**

| **Variable** | **Under 5 years**  **n = 28** | **5 years or older**  **n = 372** | **p-valor*** |
| --- | --- | --- | --- |
| **Year of diagnosis** |  |  | 0.005 |
| 2022 | 0 (0.0%) | 81 (21.8%) |  |
| 2023 | 8 (28.6%) | 97 (26.1%) |  |
| 2024 | 20 (71.4%) | 194 (52.2%) |  |
| **Male sex** | 10 (35.7%) | 173 (46.5%) | 0.327 |
| **District of origin** |  |  | 0.298 |
| Belén | 27 (96.4%) | 361 (97.0%) |  |
| San Juan | 0 (0.0%) | 9 (2.4%) |  |
| Iquitos | 1 (3.6%) | 2 (0.5%) |  |
| **Area of origin** |  |  | 0.129 |
| Urbano | 11 (39.3%) | 163 (43.8%) |  |
| Urbano-marginal | 5 (17.9%) | 114 (30.6%) |  |
| Rural | 12 (42.9%) | 95 (25.5%) |  |
| **Residence in flood-prone area** | 14 (50.0%) | 160 (43.0%) | 0,554 |
| **Symptoms** |  |  |  |
| Fever | 20 (71.4%) | 311 (83.6%) | 0.118 |
| Headache | 10 (35.7%) | 247 (66.4%) | 0.002 |
| Retro-orbital pain | 1 (3.6%) | 28 (7.5%) | 0.709 |
| Malaise | 22 (78.6%) | 190 (51.1%) | 0.005 |
| Anorexia | 3 (10.7%) | 48 (12.9%) | 1.000 |
| Asthenia | 2 (7.1%) | 36 (9.7%) | 1.000 |
| Dizziness | 2 (7.1%) | 84 (22.6%) | 0.058 |
| Chills | 2 (7.1%) | 133 (35.8%) | 0.001 |
| Low back pain | 2 (7.1%) | 60 (16.1%) | 0.282 |
| Musculoskeletal pain | 1 (3.6%) | 38 (10.2%) | 0.503 |
| Calf pain | 0 (0.0%) | 29 (7.8%) | 0.247 |
| Precordial pain | 0 (0.0%) | 5 (1.3%) | 1.000 |
| Myalgia | 4 (14.3%) | 61 (16.4%) | 1.000 |
| Arthralgia | 2 (7.1%) | 37 (9.9%) | 1.000 |
| Rash | 2 (7.1%) | 14 (3.8%) | 0.310 |
| Nausea/vomiting | 6 (21.4%) | 86 (23.1%) | 1.000 |
| Abdominal pain | 3 (10.7%) | 45 (12.1%) | 1.000 |
| Constipation | 1 (3.6%) | 6 (1.6%) | 0.401 |
| Petechiae | 0 (0.0%) | 4 (1.1%) | 1.000 |
| Epistaxis | 0 (0.0%) | 2 (0.5%) | 1.000 |
| Hematuria | 0 (0.0%) | 2 (0.5%) | 1.000 |
| Oliguria | 1 (3.6%) | 3 (0.8%) | 0.253 |
| Gastrointestinal hemorrhage | 0 (0.0%) | 1 (0.3%) | 1.000 |
| Cough | 6 (21.4%) | 47 (12.6%) | 0.240 |
| Dyspnea | 0 (0.0%) | 14 (3.8%) | 0.612 |
| Diarrhea | 4 (14,3%) | 44 (11.8%) | 0.761 |
| Blood pressure |  |  | 0.844 |
| Hypotension | 1 (3.6%) | 15 (4.0%) |  |
| Normotension | 24 (85.7%) | 296 (79.6%) |  |
| Hypertension | 3 (10.7%) | 61 (16.4%) |  |
| Heart rate |  |  | 0.476 |
| Bradycardia | 0 (0.0%) | 10 (2.7%) |  |
| Normal heart rate | 28 (100%) | 341 (91.7%) |  |
| Tachycardia | 0 (0.0%) | 21 (5.6%) |  |
| Malnutrition | 14 (50.0%) | 55 (14.8%) | <0.001 |
| **Duration of illness greater than 3 days** | 11 (39.3%) | 274 (73.7%) | <0.001 |
| **Identified serovar** |  |  |  |
| *Varillal* | 24 (85.7%) | 316 (84.9%) | 1.000 |
| *Icterohemorraghiae* | 2 (7.1%) | 41 (11.0%) | 0.754 |
| *Bataviae* | 0 (0.0%) | 3 (0.8%) | 1.000 |
| *Bratislava* | 3 (10.7%) | 28 (7.5%) | 0.468 |
| *Autumnalis* | 0 (0.0%) | 5 (1.3%) | 1.000 |
| *Hurstbridge* | 0 (0.0%) | 47 (12.6%) | 0.060 |
| *Panama* | 2 (7.1%) | 5 (1.3%) | 0.080 |
| *Canicola* | 0 (0.0%) | 4 (1.1%) | 1.000 |
| *Coxi* | 0 (0.0%) | 1 (0.3%) | 1.000 |
| *Australis* | 1 (3.6%) | 7 (1.9%) | 0.443 |
| *Javanica* | 0 (0.0%) | 1 (0.3%) | 1.000 |
| *Cynopteri* | 1 (3.6%) | 2 (0.5%) | 0.196 |
| *Hardjo* | 0 (0.0%) | 1 (0.3%) | 1.000 |
| *Djasiman* | 0 (0.0%) | 1 (0.3%) | 1.000 |
| *Copenhag* | 1 (3.6%) | 12 (3.2%) | 1.000 |

Notes:

- Data are presented as n (%).
- Percentages were calculated by column according to the total number in each age group.
- The p value was calculated using Fisher’s exact test to compare proportions between the <5 years and ≥5 years groups.
- Variables with multiple categories are presented in separate rows, and the p value corresponds to the overall comparison of the variable between groups.
- n: number of patients.
