# Supplementary material for: Clinical and epidemiological characteristics of leptospirosis in patients under and over 5 years of age in primary health centers in the Peruvian Amazon, 2022–2024
Source: PLoS Negl Trop Dis. 2026 Jun 25;20(6):e0013473. doi: 10.1371/journal.pntd.0013473 (PMC13421768; doi:10.1371/journal.pntd.0013473)
Supplement: Attachment — Submitted filename: Response to Reviewers - May 27 2026.docx [file pntd.0013473.s009.docx]

**PLOS Neglected Tropical Diseases**

*Manusctipt PNTD-D-25-01422R1*

| **Manuscript title** | Differential profile of Leptospirosis in patients under and over 5 years of age in health centers of the Peruvian Amazon, 2022-2024 |
| --- | --- |
| **Journal** | PLOS Neglected Tropical Diseases |
| **Resubmission deadline** | May 28, 2026, 11:59 p.m. |

**Reviewers' comments:** Reviewer's Responses to Questions

**Key Review Criteria Required for Acceptance?**
As you describe the new analyses required for acceptance, please consider the following:

**Methods**
-Are the objectives of the study clearly articulated with a clear testable hypothesis stated?

-Is the study design appropriate to address the stated objectives?

-Is the population clearly described and appropriate for the hypothesis being tested?

-Is the sample size sufficient to ensure adequate power to address the hypothesis being tested?

-Were correct statistical analysis used to support conclusions?

-Are there concerns about ethical or regulatory requirements being met?

Reviewer #1: The objective is now clearly stated and appropriately framed as descriptive and comparative; however, a formal testable hypothesis is still not explicitly defined. The study is better positioned as exploratory rather than hypothesis-driven.

Yes. The retrospective cross-sectional design is appropriate for the stated descriptive objective of characterizing clinical and epidemiological patterns in a primary care setting, although it remains limited for causal inference.

Yes. The study population is now clearly defined, including the total suspected cases and selection process, and is appropriate for the descriptive aims. However, representativeness is influenced by primary care–based sampling and uneven distribution across centers.

No. While the overall sample size (n=400) is adequate for descriptive purposes, the subgroup of children under 5 years (n=28) remains underpowered for robust comparative or inferential analyses.

Partially. The statistical approach has improved, including the use of Fisher’s exact test and explicit acknowledgment of exploratory analyses without multiple testing correction. However, results should still be interpreted cautiously given small cell counts, multiple comparisons, and limited power.

No. Ethical approval, data anonymization, and waiver of informed consent are clearly described and appropriate for a retrospective study using routine clinical records.

**Response: We thank the reviewer for this careful and constructive assessment. We agree that the study is better framed as descriptive and exploratory rather than hypothesis-driven. To avoid implying a confirmatory analytical design, we have clarified in the Methods section that no a priori confirmatory hypothesis was tested. We also added that no formal sample size calculation was performed because the study included all eligible MAT-confirmed cases with sufficient information during the study period.**

**In response to the reviewer’s concerns regarding the small number of children under 5 years and limited statistical power, we further clarified that subgroup comparisons, particularly those involving children under 5 years, should be interpreted as exploratory. We also strengthened the Statistical analysis section to state that p-values were interpreted as descriptive measures of statistical evidence rather than confirmatory tests of association, and that no causal or definitive age-related inferences were made.**

**These changes were made in the Methods section under Study design, Population and sample, and Statistical analysis.**

**Changes made in the manuscript**

**Study design: “The study was designed as a descriptive and exploratory analysis; therefore, no a priori confirmatory hypothesis was tested.”**

**Population and sample: “No formal sample size calculation was performed because the study included all eligible MAT-confirmed cases with sufficient information during the study period. Therefore, subgroup comparisons, particularly those involving children under 5 years of age, should be interpreted as exploratory.”**

**Statistical analysis: “Given that these analyses were exploratory and multiple variables were assessed, no formal correction for multiple testing was applied. Accordingly, p-values were interpreted as descriptive measures of statistical evidence rather than confirmatory tests of association, and no causal or definitive age-related inferences were made.”**

**Results**

-Does the analysis presented match the analysis plan?

-Are the results clearly and completely presented?

-Are the figures (Tables, Images) of sufficient quality for clarity?

Reviewer #1: Yes, overall. The analyses presented are now consistent with the stated plan, including descriptive summaries and exploratory bivariate comparisons. The authors appropriately clarified that comparisons are exploratory and avoided overuse of multivariate modeling.

Yes, for the most part. The results are clearly structured, with appropriate separation between descriptive findings and exploratory comparisons. The inclusion of supplementary tables improves transparency. However, interpretation should remain cautious given the small pediatric sample and exploratory nature of the analyses.

Tables are clear and informative. Figures have improved (e.g., inclusion of a GIS-based map), but some visual elements, particularly the density map, could still be refined to better align with scientific cartographic standards and avoid potential misinterpretation.

**Response: We thank the reviewer for this positive and constructive assessment. We agree that the Results section should continue to emphasize the exploratory nature of the comparisons, particularly because of the small number of children under 5 years. To address this, we revised the Results text to further clarify that p-values should be interpreted descriptively rather than as confirmatory evidence of age-related differences.**

**We also reviewed the tables for consistency and corrected a formatting error in Table 6 regarding the variable “duration of illness >3 days” among children under 5 years. The corrected value is now presented as 15 (53.6%). In addition, we refined the wording of the bivariate comparison paragraph to avoid overinterpretation of statistically significant findings.**

**Regarding the figures, we revised the Figure 3 title/legend to clarify that the kernel density surface represents the concentration of confirmed cases by reporting primary health center and should not be interpreted as the residential location or precise site of infection of individual patients.**

**Changes made in the manuscript**

**Results section: “Due to the small number of children under 5 years and the exploratory nature of these comparisons, p-values should be interpreted descriptively rather than as confirmatory evidence of age-related differences.”**

**Table 6: The value for “duration of illness >3 days” among children under 5 years was corrected to 15 (53.6%).**

**Figure 3 legend: “The density surface represents the concentration of confirmed cases according to the reporting primary health center and should not be interpreted as the residential location or precise site of infection of individual patients.”**

**Conclusions**

-Are the conclusions supported by the data presented?

-Are the limitations of analysis clearly described?

-Do the authors discuss how these data can be helpful to advance our understanding of the topic under study?

-Is public health relevance addressed?

Reviewer #1: Yes, largely. The conclusions are now more appropriately aligned with the descriptive and exploratory nature of the study, and major overinterpretations have been addressed.

Yes. The limitations section has been substantially improved and now addresses key methodological, statistical, and contextual constraints, although a few additional points could still be more explicitly stated.

Yes. The manuscript clearly highlights the relevance of outpatient and pediatric presentations in a hyperendemic setting and frames the findings as contributing to improved clinical awareness and future research directions.

Yes. The study effectively emphasizes implications for primary care recognition, surveillance, and management of leptospirosis in vulnerable populations within endemic regions.

**Response:**

**We thank the reviewer for this positive assessment. We are pleased that the revised conclusions were considered more appropriately aligned with the descriptive and exploratory nature of the study. In response to the reviewer’s suggestion that some limitations could be stated more explicitly, we further revised the Limitations section to address two additional points.**

**First, we added a statement on potential symptom ascertainment bias in young children, noting that subjective symptoms may be more difficult to report and document in this age group and may depend more heavily on caregiver report and clinician interpretation. Second, we added a statement on diagnostic constraints related to MAT, clarifying that single-sample MAT results may limit the distinction between recent infection, previous exposure, and cross-reactive serological responses, and that MAT serogroup reactivity should not be interpreted as definitive identification of the infecting serogroup.**

**We also made a minor revision to the conclusion to maintain a cautious public health message while emphasizing the need to strengthen clinical suspicion, diagnostic capacity, and epidemiological surveillance in primary care.**

**Changes made in the manuscript**

**Limitations section: “Symptom ascertainment may also have differed by age group, as young children may have limited ability to report subjective symptoms such as headache, myalgia, chills, dizziness, or abdominal pain, making documentation more dependent on caregiver report and clinician interpretation.”**

**Limitations section: “Although MAT is the reference serological method for leptospirosis surveillance, the use of single-sample MAT results in this retrospective setting may have limited the ability to distinguish recent infection from previous exposure or cross-reactive serological responses, and MAT serogroup reactivity should not be interpreted as definitive identification of the infecting serogroup.”**

**Conclusion: “These findings support the need to strengthen clinical suspicion, diagnostic capacity, and epidemiological surveillance for pediatric leptospirosis in primary care.”**

**Editorial and Data Presentation Modifications?**

Use this section for editorial suggestions as well as relatively minor modifications of existing data that would enhance clarity. If the only modifications needed are minor and/or editorial, you may wish to recommend “Minor Revision” or “Accept”.

Reviewer #1: The manuscript has been substantially improved in clarity, structure, and alignment between methods, results, and conclusions. Remaining issues are mostly minor and editorial. Terminology should be fully standardized throughout the text (e.g., consistent use of “serovar/serogroup” and uniform age-group definitions). Some sentences could benefit from minor language polishing to improve readability and precision in English. Tables are generally clear, but minor formatting adjustments (e.g., consistent reporting of percentages and reference categories) would further enhance clarity. The revised figures represent an improvement, particularly the inclusion of a GIS-based map; however, minor refinements in cartographic presentation (e.g., legend clarity and symbology) could still improve interpretability. Overall, only minor editorial adjustments are needed to finalize the manuscript for publication.

**Response:**

**We thank the reviewer for this positive assessment and for identifying the remaining editorial issues. We carefully revised the manuscript to improve consistency, readability, and data presentation.**

**First, we standardized terminology throughout the manuscript by consistently using “MAT serogroup reactivity” when referring to MAT results, rather than implying definitive serovar identification. We also standardized the age-group terminology, using “children under 5 years” and “patients aged 5 years or older” in the narrative text, and “<5 years” and “≥5 years” in tables.**

**Second, we performed minor English language polishing throughout the manuscript to improve readability and precision, while preserving the descriptive and exploratory framing of the study.**

**Third, we reviewed the tables for formatting consistency, including percentage reporting, age-group labels, p-value presentation, and category formatting.**

**Finally, we refined the Figure 3 title and legend to improve cartographic clarity. Specifically, we clarified that the kernel density surface represents the concentration of confirmed cases according to the reporting primary health center and should not be interpreted as the residential location or precise site of infection of individual patients.**

**Changes made in the manuscript:**

**Terminology: “MAT serogroup reactivity” was used consistently throughout the manuscript.**

**Age-group labels: Narrative text was standardized as “children under 5 years” and “patients aged 5 years or older.” Tables were standardized as “<5 years” and “≥5 years.”**

**Table formatting: Percentages, p-values, and age-group headers were reviewed for consistency. The Table 6 value for “duration of illness >3 days” among children under 5 years was corrected to 15 (53.6%).**

**Figure 3 legend: “The density surface represents the concentration of confirmed cases according to the reporting primary health center and should not be interpreted as the residential location or precise site of infection of individual patients.”**

**Summary and General Comments**

Use this section to provide overall comments, discuss strengths/weaknesses of the study, novelty, significance, general execution and scholarship. You may also include additional comments for the author, including concerns about dual publication, research ethics, or publication ethics. If requesting major revision, please articulate the new experiments that are needed.

Reviewer #1: The revised version shows substantial improvement compared to the original submission. The authors have appropriately reframed the study as descriptive and exploratory, clarified the study population and denominators, improved the statistical approach (including the use of Fisher’s exact test and clearer distinction between descriptive and inferential analyses), and significantly strengthened the Limitations section. Importantly, conclusions are now more proportionate to the data and avoid prior overinterpretation.

The main strengths of the study include the use of laboratory-confirmed cases, the multi-year dataset, and the focus on primary care settings, which are often underrepresented in leptospirosis research. The emphasis on young children, despite the small sample size, highlights an important gap in the literature and provides a basis for future investigation.

Remaining weaknesses are relatively minor and largely editorial. These include the need for full standardization of terminology, minor improvements in clarity and English language, and small refinements in table formatting and figure presentation. While the limitations have been substantially improved, a few additional points (e.g., symptom ascertainment bias in young children and diagnostic constraints related to MAT) could be stated more explicitly, although these do not materially compromise the overall interpretation.

I suggest revising the title to better reflect the descriptive and exploratory nature of the study and to avoid implying strong differential patterns that are not fully supported by the data. A more neutral and precise formulation would improve alignment between the title and the study design. My suggestion: "Clinical and epidemiological characteristics of leptospirosis in patients under and over 5 years of age in primary health centers in the Peruvian Amazon, 2022–2024"

**Response: We thank the reviewer for this thoughtful and encouraging overall assessment of the revised manuscript. We appreciate the recognition of the improvements made in the framing of the study, clarification of the study population, statistical approach, limitations, and proportional interpretation of the findings.**

**We agree that the remaining issues are mainly editorial and related to clarity, consistency, and presentation. Accordingly, we standardized terminology throughout the manuscript, particularly the use of “MAT serogroup reactivity,” and harmonized the age-group definitions across the text and tables. We also performed minor English language polishing and reviewed the tables for formatting consistency.**

**In response to the reviewer’s recommendation, we further strengthened the Limitations section by explicitly addressing potential symptom ascertainment bias in young children and diagnostic constraints related to MAT. In addition, we revised the Figure 3 title and legend to improve cartographic clarity and avoid misinterpretation of the kernel density map.**

**Finally, we agree with the reviewer’s suggestion that the title should better reflect the descriptive and exploratory nature of the study. We therefore revised the manuscript title as suggested.**

**Changes made in the manuscript:**

**Title: “Clinical and epidemiological characteristics of leptospirosis in patients under and over 5 years of age in primary health centers in the Peruvian Amazon, 2022–2024”**

**Terminology: “MAT serogroup reactivity” was used consistently throughout the manuscript.**

**Limitations: “Symptom ascertainment may also have differed by age group, as young children may have limited ability to report subjective symptoms such as headache, myalgia, chills, dizziness, or abdominal pain, making documentation more dependent on caregiver report and clinician interpretation.”**

**Limitations: “Although MAT is the reference serological method for leptospirosis surveillance, the use of single-sample MAT results in this retrospective setting may have limited the ability to distinguish recent infection from previous exposure or cross-reactive serological responses, and MAT serogroup reactivity should not be interpreted as definitive identification of the infecting serogroup.”**

**Figure 3: The title and legend were revised to clarify that the density surface represents the concentration of confirmed cases by reporting primary health center and should not be interpreted as the residential location or precise site of infection of individual patients.**

**Figure resubmission:**

While revising your submission, we strongly recommend that you use PLOS’s NAAS tool (<https://ngplosjournals.pagemajik.ai/artanalysis>) to test your figure files. NAAS can convert your figure files to the TIFF file type and meet basic requirements (such as print size, resolution), or provide you with a report on issues that do not meet our requirements and that NAAS cannot fix.

After uploading your figures to PLOS’s NAAS tool - <https://ngplosjournals.pagemajik.ai/artanalysis>, NAAS will process the files provided and display the results in the "Uploaded Files" section of the page as the processing is complete. If the uploaded figures meet our requirements (or NAAS is able to fix the files to meet our requirements), the figure will be marked as "fixed" above. If NAAS is unable to fix the files, a red "failed" label will appear above. When NAAS has confirmed that the figure files meet our requirements, please download the file via the download option, and include these NAAS processed figure files when submitting your revised manuscript.

**Reproducibility:**

To enhance the reproducibility of your results, we recommend that authors of applicable studies deposit laboratory protocols in [protocols.io](http://protocols.io/), where a protocol can be assigned its own identifier (DOI) such that it can be cited independently in the future. Additionally, PLOS ONE offers an option to publish peer-reviewed clinical study protocols. Read more information on sharing protocols at <https://plos.org/protocols?utm_medium=editorial-email&utm_source=authorletters&utm_campaign=protocols>

**Response: In addition, we reviewed the revised figure files in accordance with PLOS figure submission recommendations and refined the Figure 3 title and legend to improve interpretability. Because this was a retrospective study based on routinely collected clinical and epidemiological records, no new laboratory protocol was developed for deposition in protocols.io. Leptospirosis confirmation was based on the Microscopic Agglutination Test (MAT), an established diagnostic technique approved and performed by the Peruvian National Institute of Health.**
