# Supplementary material for: Clinical and epidemiological characteristics of leptospirosis in patients under and over 5 years of age in primary health centers in the Peruvian Amazon, 2022–2024
Source: PLoS Negl Trop Dis. 2026 Jun 25;20(6):e0013473. doi: 10.1371/journal.pntd.0013473 (PMC13421768; doi:10.1371/journal.pntd.0013473)
Supplement: Attachment — Submitted filename: Comments_PNTD-D-25-01422 REPLY TO COMMENTS 13 March 2026.docx [file pntd.0013473.s008.docx]

The authors present a retrospective, primary care-based study describing the clinical and epidemiological characteristics of MAT-confirmed leptospirosis cases in the district of Belén, Loreto, with a particular focus on comparisons between patients under and over five years of age. The manuscript addresses an important and underexplored population in a hyperendemic Amazonian setting and provides valuable descriptive data derived from routine health services, which are rarely reported in the literature. Strengths of the study include the use of laboratoryconfirmed cases, the multi-year observation period, and the emphasis on outpatient presentations in vulnerable communities, all of which enhance the relevance of the work for public health practice in endemic regions. The has the potential to contribute meaningful insights into age-related differences in leptospirosis at the primary care level. However, I have several important methodological and interpretative concerns that need to be carefully addressed and revised before the manuscript can be considered for acceptance. I strongly suggest that the next version include numbered lines to facilitate the review process.

# Major Comments

1) The most critical methodological concern relates to the use of binary logistic regression with “being under 5 years of age” as the outcome variable, based on only 28 children under five. Modeling age group as a dependent variable is conceptually problematic, as age is not a biological or clinical outcome but an inherent characteristic. As a result, several reported associations (e.g., duration of illness increasing the likelihood of belonging to the <5 group) lack biological plausibility and may reflect statistical artifacts rather than meaningful epidemiological relationships.

Moreover, the very small number of events (<5 years) substantially limits the reliability of multivariate modeling. Even with only two predictors retained, the analysis is highly susceptible to overfitting, unstable odds ratios, and inflated effect sizes. The extremely strong association observed for underweight status (OR = 0.051) further suggests model instability and/or issues with variable coding and reference categories.

The authors should reconsider the use of multivariate regression altogether. At minimum, the regression model should be removed or reframed, and the study should be clearly positioned as descriptive and exploratory, with cautious interpretation of bivariate associations only.

**Our Response: We thank the reviewer for this important methodological observation. We agree that modeling “being under 5 years of age” as the dependent variable is conceptually problematic in this context, since age group is an inherent characteristic rather than a clinical outcome. We also agree that the very small number of children under 5 years of age substantially limits the reliability of multivariable modeling and may result in unstable estimates and difficult interpretation.**

**Action Taken: In response to this concern, we removed the multivariable logistic regression analysis and deleted Table 7 and all corresponding regression results from the revised manuscript. We repositioned the study as a descriptive and exploratory analysis, with emphasis on age-stratified descriptive patterns and exploratory bivariate comparisons only. We also revised the Methods, Results, and Discussion sections to ensure that findings are presented cautiously and interpreted as hypothesis-generating rather than confirmatory.**

**Location: Methods, Statistical Analysis; Results and Discussion sections; Table 7 removed from the revised manuscript.**

2) The Methods section states that all variables, including duration of illness, were treated as categorical. However, in the multivariate model, duration of illness is clearly treated as a continuous quantitative variable, with interpretation “for each additional day of illness.”

This inconsistency undermines the transparency and reproducibility of the analysis. Furthermore, duration of illness was not significant in the bivariate analysis (p = 0.32) but became highly significant in the multivariate model, which is not adequately explained and raises concerns about analytical coherence.

The authors must clarify how duration of illness was coded and analyzed, ensure consistency between methods and results, and justify the biological and epidemiological meaning of this association. Without clarification, this result should not be retained.

**Our Response: We thank the reviewer for identifying this important inconsistency. We agree that the previous presentation of duration of illness was not sufficiently consistent between the Methods and the regression analysis, and that this undermined the transparency of the analysis.**

**Action Taken: We removed the multivariable logistic regression analysis and deleted all corresponding results from the revised manuscript. Accordingly, the inconsistency related to treating duration of illness as a continuous variable in the regression model no longer applies. In the revised manuscript, time from symptom onset to consultation is described consistently as a categorical variable in the descriptive analysis and, for exploratory bivariate comparison, as a dichotomized variable (≤3 vs >3 days), as now clearly stated in the Methods and reflected in the Results.**

**Location: Methods, Statistical Analysis; Results section; Table 7 removed from the revised manuscript.**

3) Nutritional status is treated using BMI categories (underweight, normal, overweight) in children under five. This is problematic, as BMI is not the recommended anthropometric indicator for this age group. International standards (e.g., WHO) recommend weight-for-height, weight-forage, or height-for-age Z-scores.

The use of BMI without justification may introduce classification bias and weakens the validity of conclusions regarding malnutrition and leptospirosis in young children.

The authors should justify the use of BMI in children under five or, preferably, reanalyze nutritional status using age-appropriate WHO growth indicators.

**Our Response: We thank the reviewer for this important observation. We agree that nutritional assessment in children under 5 years of age should be based on age-appropriate anthropometric standards and that BMI alone is not the preferred indicator for this age group.**

**Action Taken: We revised the manuscript to clarify that nutritional status was classified using age-specific anthropometric criteria in accordance with World Health Organization recommendations. In children under 5 years of age, undernutrition was defined using weight-for-height Z score (WHZ) < −2 SD. In children and adolescents aged 5–18 years, undernutrition was defined using BMI-for-age Z score (BAZ) < −2, and in adults (≥19 years), undernutrition was defined as BMI <18.5 kg/m². We also revised the Results and Discussion to ensure that nutritional findings in children under 5 years are interpreted within this age-specific anthropometric framework and with appropriate caution.**

**Location: Methods, Population and sample subsection; Results and Discussion sections.**

4) The manuscript reports statistically significant associations between age group and specific “species/serovars” (Panama and Hurstbridge). However:
   a) The absolute numbers are extremely small (e.g., 2 cases of Panama in children <5).
   b) Pearson’s chi-square test is used despite small cell counts, where Fisher’s exact test would be more appropriate.
   c) Multiple serovars and symptoms are tested without adjustment for multiple comparisons, increasing the risk of false-positive findings.
   d) MAT-based serovar attribution is inherently limited by cross-reactivity, which the authors acknowledge but still interpret as evidence of “acute infection caused by a specific serovar.”

Serovar-specific conclusions should be clearly framed as exploratory and hypothesis-generating only. Strong statements regarding virulence, transmission patterns, or vaccine relevance are not supported by the data and should be substantially softened.

**Our Response: We thank the reviewer for this important methodological comment. We agree that the serogroup-specific findings are based on very small absolute counts, that Fisher’s exact test is more appropriate for sparse data, and that MAT serogroup reactivity should be interpreted cautiously given cross-reactivity and the exploratory nature of these comparisons. We also agree that these results should not be presented as evidence of organism-level attribution, virulence, transmission patterns, or vaccine relevance.**

**Action Taken: We revised the manuscript in several ways. First, we replaced Pearson’s chi-square test with Fisher’s exact test for sparse bivariate comparisons. Second, we revised the terminology throughout the manuscript to refer to MAT serovar/serogroup reactivity rather than “species,” and we removed statements implying organism-level attribution or acute infection caused by a specific serovar. Third, we substantially softened the Results and Discussion to state explicitly that the observed serogroup-related differences are based on very small numbers and should be interpreted as exploratory and hypothesis-generating only. We also clarified that MAT reflects serologic reactivity rather than definitive organism-level characterization. Finally, we added text indicating that no formal multiple-testing correction was applied because the analyses were exploratory, and that p-values should therefore be interpreted cautiously; the full set of exploratory bivariate comparisons is now reported in Supplementary Table S2.**

**Location: Methods, Statistical Analysis subsection; Results, serogroup reactivity paragraph; Discussion, microbiological interpretation paragraph; Supplementary Table S2.**

5) Several symptoms compared between <5 and ≥5 years (e.g., headache, myalgia, musculoskeletal pain) depend on subjective reporting, which is inherently limited in young children. Differences observed may reflect reporting and ascertainment bias rather than true biological differences.

This limitation is not sufficiently discussed and undermines the interpretation of age-specific clinical profiles. The Discussion should explicitly acknowledge differential symptom reporting capacity by age and avoid implying pathophysiological differences based solely on these comparisons.

**Our Response: We thank the reviewer for this important observation. We agree that several symptom comparisons, particularly for subjective symptoms such as headache, myalgia, and musculoskeletal pain, may be affected by age-related differences in reporting capacity and clinical ascertainment. We also agree that these findings should not be interpreted as evidence of underlying pathophysiological differences based solely on retrospective symptom comparisons.**

**Action Taken: We revised the Discussion to explicitly acknowledge that subjective symptoms are more difficult to identify, report, and document in very young children, especially pre-verbal children, and that this may have influenced the observed age-group differences. We also softened the interpretation of symptom-related findings throughout the manuscript and framed these comparisons as descriptive, exploratory, and hypothesis-generating rather than as evidence of true biological differences.**

**Location: Discussion, symptom interpretation paragraphs; Limitations section.**

6) All cases were mild, with no hospitalizations or deaths, as expected from a primary care-based study. However, many conclusions are written broadly, implying relevance to leptospirosis in general rather than outpatient, mild disease only.

The scope of inference should be more clearly restricted throughout the Abstract, Discussion, and Conclusions to mild leptospirosis managed at the primary care level.

**Our Response: We thank the reviewer for this important comment. We agree that, because the study was based on confirmed cases managed at first-level outpatient health facilities, the scope of inference should be restricted to predominantly mild leptospirosis evaluated in primary care rather than leptospirosis in general.**

**Action Taken: We revised the manuscript throughout to narrow the scope of interpretation accordingly. Specifically, we clarified in the Abstract, Discussion, and Conclusions that the findings refer to MAT-confirmed leptospirosis cases evaluated at primary health care centers and therefore represent a predominantly mild outpatient clinical spectrum. We also softened statements that could imply generalizability to hospitalized or severe cases and added explicit wording noting that patients with warning signs or suspected severe disease may have been referred to higher levels of care and are likely underrepresented in this series.**

**Location: Abstract; Discussion; Conclusions; Limitations section.**

**Minor Comments**

# Title and Abstract

**Page 1, Title**: The title refers to “2023–2024,” while the Methods and Results include 2022– 2024. This inconsistency should be corrected.

**Our Response: We thank the reviewer for carefully identifying this discrepancy. The year range indicated in the original title was due to a typographical oversight. We have corrected the title to reflect the full study period (2022–2024), consistent with the Methods and Results sections.**

**Action Taken: Title revised to: “Differential profile of leptospirosis in patients younger and older than 5 years in primary health care centers in the Peruvian Amazon, 2022–2024.”**

**Location: Page 1 (Title).**

**Page 2, Abstract**: The phrase “each additional day of illness was associated with an increased likelihood of belonging to this age group” is conceptually unclear and should be rephrased or removed.

The abstract should be adapted according to all the suggestions made throughout the text.

The phrase “each additional day of illness was associated with an increased likelihood of belonging to this age group” is conceptually unclear and should be rephrased or removed. The abstract should be adapted according to all the suggestions made throughout the text.

**Response: We thank the reviewer for this helpful observation. We agree that the referenced sentence was conceptually unclear and did not improve interpretation of the findings.**

**Action taken: We have removed this sentence from the Abstract as suggested. In addition, we revised the entire Abstract to ensure it accurately reflects all modifications made throughout the manuscript in response to the reviewers’ comments.**

**Location in manuscript: Page 2 (Abstract).**

# Introduction

The introduction is too dense and descriptive in places and could be more argument-driven: it lists facts but does not sufficiently explain why under-5s deserve a dedicated analysis beyond “less studied.”

The transition from Peru burden to age differences is a bit abrupt; you could add one bridging sentence explaining why the high-burden setting makes age-stratified outpatient profiles clinically important.

**Response: Thank you for this constructive feedback. We agree that the original Introduction was overly descriptive in parts and did not sufficiently articulate the rationale for an age-stratified analysis focused on children younger than five years. We revised the Introduction to be more argument-driven by streamlining background content and by explicitly explaining why under-5 children merit focused evaluation in endemic, primary-care settings, including diagnostic recognition challenges in early childhood, the frequent absence of classic adult-oriented features that typically prompt suspicion, and evidence suggesting differences in severity patterns and clinical presentation across age groups.**

**Action Taken: We edited the Introduction for clarity and concision, reduced redundant descriptive content, and reorganized the narrative to strengthen the study rationale. We also added a bridging sentence linking the high-burden context in Peru to the clinical relevance of age-stratified outpatient profiles in primary care. Finally, we expanded the paragraph on age-related differences to better justify the under-5 focus using supporting evidence and emphasizing outpatient diagnostic implications.**

**Location: Page 4-5**

**Page 4:** The phrase “Among the most affected species, rodents are considered chronic carriers…” This is conceptually confusing: rodents are not “most affected species,” they are key reservoir hosts. On the other hand, dogs, pigs, and cattle can both be carriers and suffer the consequences of the infection. A more appropriate reference should be included here.

**Response: We thank the reviewer for this clarification. We agree that the phrase “most affected species” was conceptually imprecise and could be misinterpreted. We revised the sentence to describe rodents and domestic animals in terms of their role as reservoirs and sources of human exposure, rather than implying they are the “most affected” by disease.**

**Action Taken: We removed the wording “Among the most affected species, rodents are considered chronic carriers…” and rewrote the passage to state that transmission occurs through exposure to urine from wild or domestic animals, particularly rodents, dogs, pigs, and cattle, and clarified that Leptospira colonizes the renal tubules of reservoir hosts and is shed in urine, contaminating soil and water.**

**Location: Page 4**

**Page 4 -5:** The section on pediatric vs adult differences is broadly accurate, but it risks sounding definitive. “Some studies suggest…” is good. However, phrases like “may vary significantly” could be supported by specifying which outcomes differ (e.g., jaundice, renal failure, mortality in hospitalized series). Add a sentence about outpatient vs hospitalized populations since your data are primary care.

**Response: We thank the reviewer for this helpful suggestion. We agree that the prior wording could sound overly definitive. We also agree that the age-related differences are best supported by specifying the particular outcomes reported in the literature and by clarifying that many comparisons come from hospitalized cohorts, whereas our data come from primary care.**

**Action Taken: We revised this section to use more cautious language and to specify the outcomes that have been reported to differ between children and adults in published series, including differences in jaundice, renal dysfunction, and case fatality in hospitalized cohorts. In addition, we added a sentence noting that many published descriptions and diagnostic frameworks are derived mainly from hospitalized or severe-case populations, while outpatient and primary-care leptospirosis may be underrecognized and may present earlier with milder, less specific syndromes, which can require different diagnostic approaches.**

**Location: Page 4-5**

**Page 5:** “limited number of studies that systematically compare clinical and epidemiological differences…” This is correct but could be more compelling if you specify: under-5 subgroup; primary care/outpatient context; Amazonian hyperendemic setting,; and diagnostic/triage relevance.

**Response: We thank the reviewer for this helpful suggestion. We agree that the final paragraph of the Introduction is more compelling when it explicitly states the under-5 subgroup, the outpatient and primary-care context of our data, the hyperendemic Amazonian setting, and the relevance for early recognition and triage.**

**Action Taken: We rewrote the final paragraph to incorporate these elements explicitly. The revised text now emphasizes the limited evidence comparing age groups in primary-care and outpatient populations, highlights the hyperendemic context of the Peruvian Amazon, and clarifies that our objective is to inform diagnostic suspicion and early management decisions at the first level of care through age-stratified outpatient profiles.**

**Location: Page 5**

**Page 5, final paragraph**: The objective is described as “to describe,” which matches the retrospective design. But “strengthen strategies at three levels” is aspirational and may read as too broad

The objective is descriptive, yet later analyses imply causal inference. Consider aligning wording more clearly with a descriptive/exploratory design.

**Response: We thank the reviewer for this important point. We agree that, given the retrospective design, the objective should remain clearly descriptive and that wording suggesting broad strategy strengthening could be interpreted as overly aspirational. We also agree that the Introduction should not imply causal inference.**

**Action Taken: We revised the final paragraph to keep the objective explicitly descriptive and comparative and removed language implying broad programmatic impact. We also reframed the potential contribution in more appropriate terms for an exploratory primary-care dataset by emphasizing age-stratified outpatient profiles that may help guide clinical recognition and initial decision-making, rather than implying causal effects.**

**Location: Page 5-6**

# Methods

**Page 5**, Study design: The term “multicenter” should be contextualized, as one health center accounts for 72% of cases. Potential center-level bias should be acknowledged.

**Response: We thank the reviewer for this important observation. We agree that the term “multicenter” should be contextualized given the disproportionate contribution from a single health center and that potential center-level bias should be acknowledged.**

**Action Taken: We removed the term “multicenter” from the study design description in the Methods. In addition, we added a statement in the Limitations section noting that most cases were contributed by a single health center, which may introduce center-level selection or referral bias and may limit generalizability across sites.**

**Location: Page 6 and Limitations paragraph**

**Page 5:** Given the strong emphasis on environmental exposure, flooding, and spatial vulnerability in the district of Belén, the inclusion of a study-area map would substantially strengthen the manuscript. A map showing the location of Belén within Loreto, the main rivers, and the four participating health centers would improve clarity and help contextualize the epidemiological findings, particularly the unequal contribution of cases across centers.

**Response: We appreciate the reviewer’s suggestion. Given the importance of environmental exposure, recurrent flooding, and spatial vulnerability in Belén, we agree that a study-area map strengthens the presentation and interpretation of the epidemiologic findings.**

**Action Taken: A new Figure 1 was added to the manuscript, depicting the geographic location of the study area within Loreto, the main surrounding rivers, and the four participating health centers (Belén Health Center, 6 de Octubre Health Center, 9 de Octubre Health Center, and Cardozo Health Center). In addition, the Methods section was revised to include a dedicated Study setting subsection describing the local environmental and service-delivery context relevant to leptospirosis transmission and case distribution.**

**Location: Page 6-7**

**Page 6**, **Case definition**: Clarify the rationale for using a single MAT titer ≥1:800 without paired samples, particularly in a hyperendemic area.

**Response: We agree that the rationale for using a single MAT titer threshold without paired samples should be explicitly stated, particularly in a hyperendemic setting.**

**Action Taken: We clarified the case definition in the Methods by stating that confirmation was based on a single MAT titer ≥1:800 in accordance with the Peruvian National Technical Health Standard, which specifies this cutoff for the Peruvian Amazon when paired sera are not available. We also noted that, due to the retrospective primary-care design, paired acute and convalescent samples were not routinely available. In addition, we added a limitation acknowledging that in hyperendemic settings a single high titer may be influenced by prior exposure.**

**Location: Page 8 and Limitations paragraph**

**Page 6:** In the sentence: “These conditions favor the proliferation of vectors and the transmission of infectious diseases…” Leptospirosis is not vector-borne; transmission is environmental exposure to contaminated urine/water/soil. “Vectors” could mislead readers.

**Response: We thank the reviewer for this important terminological correction. We agree that leptospirosis is not vector-borne and that the term “vectors” could mislead readers regarding transmission.**

**Action Taken: We removed the term “vectors” and revised the sentence to describe transmission more accurately as increased environmental exposure associated with flooding and unsanitary conditions. We also clarified that leptospirosis transmission in this setting is linked to contact with water or soil contaminated with urine from infected animals, particularly rodents.**

**Location: Page 6**

**Page 6, Statistical analysis**:

Clarify why Pearson’s chi-square was used instead of Fisher’s exact test for sparse data.

Explicitly state how missing data were handled.

Clarify reference categories used in the logistic regression.

**Response: We thank the reviewer for these helpful suggestions to improve the robustness and clarity of our statistical analysis. We agree that Fisher’s exact test is more appropriate for sparse data, that the handling of missing data should be explicitly stated, and that reference categories should be clarified when regression models are presented.**

**Action Taken: We re-analyzed the bivariate comparisons using Fisher’s exact test instead of Pearson’s chi-square test when sparse cell counts were present, and we revised the Statistical Analysis section accordingly. We also clarified the handling of missing data by stating that five records with insufficient data for analysis were excluded before statistical analysis; this exclusion process is additionally shown in the newly added flow diagram (Figure 2). The final analytical sample therefore included only confirmed cases with complete information for the variables analyzed. Finally, Table 7 containing the logistic regression analysis was removed from the revised manuscript, so clarification of reference categories is no longer applicable.**

**Location: Page 9-10**

# Results

**Page 7:** The Results describe a “progressive increase” and a 103.8% rise in 2024 vs 2023. However, the Results do not provide denominators or context (e.g., number tested, changes in testing practices, reporting completeness, outbreak alerts, flooding intensity, or changes in referral pathways). In a retrospective record review, increases may reflect surveillance intensity rather than true incidence. Add supporting context or report: number of suspected cases tested per year, positivity rate per year, any changes in testing/referral policies, or explicitly state this is “increase in confirmed cases identified in records,” not necessarily incidence.

Avoid causal language. For example: Replace “a progressive increase was observed” with “a higher number of confirmed cases was recorded in 2024 compared with prior years.”

**Response: We appreciate this important observation. We agree that, in a retrospective record review, temporal differences in the number of confirmed cases should not be interpreted as changes in incidence without year-specific denominators.**

**Action Taken: We revised the Results to use strictly descriptive language, indicating only that a higher number of confirmed cases was recorded in 2024 compared with prior years. We also clarified that, although the study was based on confirmed cases identified among suspected cases evaluated during the study period, the number of suspected cases was not available stratified by year; therefore, annual differences cannot be interpreted as incidence trends or yearly positivity changes.**

**Location: Page 10-11 and Limitations paragraph**

**Page 7:** One health center accounts for 72% of cases (C.S 06 de Octubre), while another contributes 1%. This makes pooled estimates heavily dependent on a single site. Without describing catchment, access, or testing behavior, this may reflect center-level ascertainment rather than epidemiology. Report per-center characteristics (even briefly) and consider adding a sensitivity description (“results were similar when restricting to the largest center”) or stratified descriptive tables by center (at least as supplementary material).

**Response: We thank the reviewer for highlighting the imbalance in case distribution across health centers and the possibility that pooled estimates may be influenced by center-level ascertainment rather than underlying epidemiology. We agree that additional center-specific information would improve the interpretability of the findings.**

**Action Taken: We revised the manuscript to provide additional center-specific context in both the Methods and Results. First, in the Study setting subsection, we added a brief description of the four participating primary health care centers, including differences in service organization and catchment characteristics that may influence the distribution of cases. Second, in the Results, we explicitly reported the distribution of confirmed cases across centers and noted the marked concentration of cases in 6 de Octubre Health Center. Third, we added a center-stratified descriptive summary as Supplementary Table S1, which presents, for each health center, the total number of confirmed cases and their distribution by age group, sex, year of diagnosis, and selected symptoms. We did not perform a formal sensitivity analysis restricted to the largest center; instead, we addressed the reviewer’s concern by providing stratified descriptive data to allow readers to assess the center-level distribution directly.**

**Location: Page 11**

**Page 7 and 9:** The authors describe “monthly prevalence” and “marked seasonal pattern,” but the data presented are case counts, not prevalence (no population denominator). The figure caption uses “Monthly prevalence,” which is incorrect. Replace “prevalence” with “monthly number of confirmed cases” (or “monthly distribution of confirmed cases”).

The statement that <5 cases were “scarce and dispersed” may simply reflect small n (28 total), limiting inference about seasonality. It should be reframed as descriptive.

**Response: We thank the reviewer for this important clarification. We agree that our data are monthly case counts and not prevalence because we do not have a population denominator, and that the term “monthly prevalence” could mislead readers.**

**Action Taken: We replaced “monthly prevalence” with “monthly distribution of confirmed cases” in the text and in the Figure 4 caption. We also revised the wording to remain descriptive for the <5 group given the small total number of cases. In addition, we clarified in the figure title and axis labeling that Figure 4 shows the total number of confirmed cases per calendar month aggregated across 2022–2024 (e.g., January totals reflect the sum of January 2022, January 2023, and January 2024), rather than prevalence or cumulative incidence over time.**

**Location: Page 11 and Figure 4**

**Page 9 (Figure 2):** The inclusion of a map is appropriate and adds value to the study; however, the current figure does not meet scientific publication standards. It appears to be based on a Google Maps screenshot with overlaid heat-style markers, which limits reproducibility and cartographic clarity. I recommend replacing it with a GIS-generated map showing the study area and the four health centers, using standardized symbology (e.g., proportional symbols), administrative boundaries, and basic cartographic elements (scale bar, north arrow, legend). This would substantially improve the scientific quality of the figure.

**Response: We thank the reviewer for this constructive critique regarding figure quality and reproducibility. We agree that the previous map, which was based on a Google Maps screenshot with heat-style overlays, did not meet scientific publication standards and limited cartographic clarity and reproducibility. We note that the comment refers to Figure 2; however, in our manuscript the map is presented as Figure 3.**

**Action Taken: We replaced the original figure with a GIS-generated map of the study area and the four participating health centers using standardized cartographic elements. The revised map includes administrative boundaries, a scale bar, a north arrow, and a detailed legend, substantially improving clarity and reproducibility. We replaced the original figure with a GIS-generated map (Figure 3) of the study area and the four participating health centers, created using Geographic Information System (GIS) software. The revised figure incorporates standardized cartographic elements, including administrative boundaries, a scale bar, a north arrow, and a legend, and now meets the expected standards for scientific publication while substantially improving clarity and reproducibility.**

**Location: Figure 3**

**Page 10:** The text says “average time … was between 3 to 6 days,” but Table 2 reports categories (0–3, 3–6, >6). That is not an “average.” Use: “The most frequent category of time from symptom onset to consultation was 3–6 days (45.8%).”

**Response: We thank the reviewer for this precise correction regarding data presentation. We agree that the term “average” is not appropriate because time-to-consultation was reported as categorical intervals.**

**Action Taken: We revised the Results text to describe the most frequent time-to-consultation category rather than an average and added the age-stratified breakdown to match Table 2.**

**Location: Page 13**

**Page 10:** Several comparisons (rural vs urban; flood-prone residence; sex distribution) are described, but it is unclear which differences were statistically tested (Table 6 lists only selected chi-square results). Either: report p-values for these key contextual variables, or explicitly state that they are descriptive and not statistically compared (and why). Also, clarify operational definitions: How was “flood-prone area” determined (self-report, address, municipal classification)?

**Response: Thank you for this important methodological observation. We agree that the manuscript should clearly distinguish between variables described descriptively and those formally examined in exploratory bivariate comparisons. We also agree that the operational definition of flood-prone residence should be stated explicitly.**

**Action Taken: We revised the Methods to clarify that residence in a flood-prone area was determined by self-report as recorded in the clinical or epidemiological forms. We also revised the Statistical Analysis and Results sections to specify that Table 6 presents only a selected subset of exploratory bivariate comparisons considered most clinically relevant for the main text. In addition, we added Supplementary Table S2, which reports the full set of exploratory comparisons, including contextual variables such as sex, district of origin, area of origin, and flood-prone residence. Variables described in Table 1 but not emphasized in the main text are therefore now clearly identified as descriptive unless shown in Supplementary Table S2.**

**Location: Page 8-9 and table 6**

**Page 10 and 11**, paragraph on “insidious onset and progressive course”: Reporting 100% of cases with identical onset and course suggests limited discriminatory value or default recording; this should be explained or removed.

**Response: We thank the reviewer for this insightful comment regarding data quality. We agree that reporting 100% of cases with the same onset and clinical course provides no discriminatory value and may reflect standardized documentation practices rather than true clinical variability.**

**Action Taken: We removed the statement regarding “insidious onset and progressive course” from the Results section to ensure that only clinically informative variables with meaningful variability are reported.**

**Location: Page 10**

**Page 11:** Table 2 shows 100% insidious onset and 100% progressive course, with no variability. This is implausible clinically and suggests default charting, poor variable definition, or that these fields were not meaningfully captured. Either remove these variables from Results, or explain why they are uniformly recorded. At minimum, acknowledge in Discussion as potential information bias.

**Response: We agree that these variables showed no variability and therefore did not provide meaningful information, likely reflecting standardized documentation rather than true clinical patterns.**

**Action Taken: We removed “type of onset” and “course of illness” from Table 2 and from the Results, and we retained only variables with meaningful variability.**

**Location: Page 13**

**Page 11, Table 1:** Percentages are sometimes reported without corresponding denominators; consider adding absolute numbers consistently.

**Response: We thank the reviewer for this comment on data presentation. We agree that reporting percentages without the corresponding absolute numbers can hinder interpretation.**

**Action Taken: We revised Table 1 to report absolute counts (n) alongside percentages (%) consistently across all variables for each age group and the total sample.**

**Location: Table 1**

**Page 11:** The Results state that symptoms like headache, chills, myalgia are more frequent in ≥5, while malaise and fever are frequent in <5. But many symptoms (headache, myalgia) are harder to ascertain in very young children. This is a major interpretation caveat and should be signposted already in Results or at least carefully in Discussion.

**Response: We thank the reviewer for this important clinical point. We agree that subjective symptoms such as headache and myalgia are more difficult to ascertain in very young children, particularly those who are pre-verbal, which may introduce differential symptom ascertainment across age groups.**

**Action Taken: We added an explicit cautionary statement in the Results noting that differences in subjective symptom frequencies between age groups should be interpreted carefully because symptom identification in children <5 years often depends on caregiver reporting.**

**Location: Page 14 and 17-18**

**Page 13:** Table 4 labels “Species,” but entries are serovars/serogroups (e.g., Icterohemorraghiae, Canicola, Autumnalis, Panama). Calling these “species” is incorrect and will undermine credibility. Please use “serovar” (or “serogroup/serovar panel reactivity by MAT”) consistently in all text and tables.

**Response: We thank the reviewer for this important taxonomic correction. We agree that the MAT panel results should not be described as “species,” as the entries correspond to serogroups, and incorrect terminology could undermine credibility.**

**Action Taken: We corrected Table 4 by replacing “Species” with “Serogroup reactivity” and revised the manuscript to use this terminology consistently throughout when referring to MAT serologic results.**

**Location: Table 4**

**Page 13:** The antibiotic patterns are reported, but the Results do not indicate: clinical severity markers, criteria guiding antibiotic choice, whether prescriptions align with national guidelines, dosing, duration, or timing relative to symptom onset. Keep descriptive, but consider adding guideline citation for pediatric management, note that treatment reflects routine practice and age-related contraindications (e.g., doxycycline in younger children).

**Response: Thank you for this important comment. We agree that our dataset does not allow evaluation of clinical severity markers, decision criteria for antibiotic choice, dosing, duration, or timing relative to symptom onset, and that our Results should remain descriptive**

**Action Taken: We revised the Results to (i) explicitly frame antibiotic use as routine practice in a primary-care/outpatient context and (ii) add a citation to the Peruvian National Technical Health Standard for leptospirosis, which outlines recommended antibiotic options for mild pediatric disease (e.g., amoxicillin or erythromycin) and adult options (including doxycycline). We also added a limitation noting that severity criteria and dosing/duration/timing were not consistently documented, so our study cannot assess guideline concordance**

**Location: Page 15-16 and Limitations paragraph**

**Page 14-15:** Table 6 reports chi-square significance for Panama and Hurstbridge. However, the absolute numbers are tiny (Panama in <5: 2 cases; Panama in ≥5: 4 cases). With such sparse counts, chi-square assumptions may be violated; Fisher’s exact test is more appropriate. Re-run these comparisons using Fisher’s exact test and report exact p-values. Additionally, consider flagging these serovar comparisons as exploratory.

**Response: We thank the reviewer for this crucial statistical correction. We agree that chi-square assumptions may be violated for sparse counts in several variables, including rare serogroup/serovar categories.**

**Action Taken: We revised the Statistical Analysis section to specify the use of Fisher’s exact test for categorical comparisons due to small cell counts and to clarify the handling of missing data. We then re-analyzed the bivariate comparisons using Fisher’s exact test and updated Table 6 to report exact p-values alongside n (%) by age group. We also removed sparse serogroup/serovar comparisons from inferential testing and framed these analyses as exploratory.**

**Location: Statystical analysis Page 9 and table 6**

**Page 14-15:** Many symptoms/variables are presented in Table 3 and Table 4, but Table 6 lists only a subset of tests and does not address the risk of false positives from multiple testing. Either apply an FDR approach (e.g., Benjamini–Hochberg) or explicitly state analyses are exploratory and interpret p-values cautiously.

**Response: Thank you for this important methodological observation. We agree that presenting multiple symptoms and contextual variables increases the risk of false-positive findings when multiple comparisons are performed. Because the aim of these analyses was exploratory and hypothesis-generating rather than confirmatory, we did not apply a formal multiple-testing correction. We therefore agree that the corresponding p-values should be interpreted cautiously.**

**Action Taken: We clarified in the Statistical Analysis section that, given the exploratory nature of the analysis and the large number of variables evaluated, no formal correction for multiple testing was applied. We also revised the Results and Discussion sections to explicitly state that the comparisons shown in Table 6 are exploratory and that their p-values should be interpreted cautiously. In addition, we clarified that Table 6 presents only selected variables highlighted in the main text, whereas the full set of exploratory bivariate comparisons is reported in Supplementary Table S2.**

**Location: Page 9 and table 6**

**Page 15:** The regression models “being <5 years” as an outcome. This is conceptually questionable (age is not a clinical outcome). The text says underweight is associated with a “higher probability” of being under 5, but the OR is 0.051, suggesting either outcome coding is reversed (≥5 coded as 1), or interpretation is flawed. The authors then attempt to explain it by describing “reduction in probability of belonging to older group,” which is confusing and nonstandard. Please explicitly state outcome coding (which group = 1), state reference categories, and rewrite interpretation clearly (or remove the model).

Bivariate duration is categorical and non-significant, but in regression duration is “quantitative” and significant. This is an important methodological inconsistency that must be corrected.

**Response: Thank you for this important methodological observation. We agree that modeling age group as the outcome created conceptual and interpretive difficulties, and that the presentation of Table 7 could be confusing.**

**Action Taken: We removed Table 7 and the corresponding regression analysis from the revised manuscript. As a result, the concerns regarding outcome coding, reference categories, interpretation of odds ratios, and the inconsistency in the handling of symptom duration in the regression model are no longer applicable.**

**Location: Page 17**

# Discussion

**Page 16, Paragraph 1:** Overstatement of “defined seasonal pattern.” The Results show monthly peaks in the ≥5 group, but you should avoid language implying robust seasonality without denominators and without controlling for surveillance intensity or testing volume. A safer formulation would be: “a seasonal clustering of cases was observed…” rather than “defined seasonal pattern.”

Causal language (“reinforces the role of the environment as a determining factor”). This is a retrospective, facility-based dataset. The Discussion should not imply causality. Suggest: “is consistent with an environmentally mediated transmission pattern”.

Interpretation for <5 group is speculative and not strongly supported. The statement that lack of seasonality in <5 could be due to reduced exposure/mobility is plausible but may simply reflect low case counts (n=28), limiting the power to detect any seasonal pattern. This limitation should be explicitly stated here.

Missing acknowledgment of ascertainment bias. Children may be taken to care differently; testing thresholds may differ; and symptom-reporting differs. These should be acknowledged when interpreting “seasonality differences.”

**Response: We thank the reviewer for these important points. We agree that, without denominators and without accounting for changes in surveillance intensity or testing volume, our results should not be presented as demonstrating robust seasonality, and the Discussion should avoid causal language. We also agree that inferences regarding the <5 group are limited by the small number of cases and that potential ascertainment and information biases should be acknowledged.**

**Action Taken: We revised the Discussion to replace definitive wording (“defined seasonal pattern”) with a more conservative description (“seasonal clustering of cases”), removed causal phrasing, and explicitly noted that the absence of a clear seasonal pattern in children <5 years should be interpreted cautiously due to the small sample size (n=28). We also added discussion of potential ascertainment and information biases (healthcare-seeking behavior, diagnostic thresholds, and symptom reporting differences in young children) that could affect apparent age-specific differences.**

**Location: Page 17**

**Page 16, Paragraph 2:** This paragraph imports statistical conclusions too strongly. “Identified as significant findings” reads as confirmatory, but the analysis has major limitations (small n, multiple comparisons, questionable regression outcome).

Internal inconsistency about duration of illness. In the Results, duration categories were not significant in bivariate analysis, yet the Discussion treats “0–3 days” as a key significant finding; this is not well supported.

The regression is conceptually problematic. Modeling “being <5 years” as an outcome should not be treated as evidence of “factors” associated with pediatric leptospirosis. This paragraph should avoid language implying independent predictors.

BMI measure in <5 is questionable. As discussed, BMI is not ideal for children under five without WHO-standard z-scores. This must be acknowledged before drawing conclusions about malnutrition and vulnerability. Reframe as descriptive/hypothesis-generating.

**Response: We thank the reviewer for this important point. We agree that the previous wording was overly confirmatory and could be misinterpreted as implying independent “factors” associated with pediatric leptospirosis. We also agree that interpreting “being <5 years” as an outcome in regression is conceptually problematic in this context and that conclusions should remain descriptive and hypothesis-generating given the small pediatric sample size and multiple comparisons. Finally, we acknowledge the concern regarding anthropometric classification in children under 5 years of age and have clarified the age-appropriate WHO-based standard used for nutritional assessment.**

**Action Taken: We revised the Discussion paragraph to remove causal or confirmatory language (e.g., “significant findings,” “factors,” and “predictors”), align the interpretation with the exploratory bivariate comparisons reported in the Results, and frame the findings as descriptive and hypothesis-generating with explicit caution regarding small sample size, multiple comparisons, and potential ascertainment bias. We also removed the regression analysis from the manuscript, thereby eliminating the conceptual concerns related to modeling age group as an outcome. In addition, we revised the Methods section to specify the anthropometric criteria used to classify nutritional status according to age group: in children under 5 years, undernutrition was defined using weight-for-height Z score (WHZ) < −2 SD; in children and adolescents aged 5–18 years, undernutrition was defined using BMI-for-age Z score (BAZ) < −2; and in adults (≥19 years), undernutrition was defined as BMI <18.5 kg/m². The Discussion was further revised to clarify that nutritional findings in children under 5 years should be interpreted within this age-specific anthropometric framework and not as evidence of causal vulnerability.**

**Location: Methods Page 9 and Discussion page 17**

**Page 17, Paragraph 3:** “Most relevant finding” is a value judgment and should be backed by a clinical rationale or softened.

Symptom comparability issue is missing. Differences in headache/myalgia/chills may reflect children’s inability to report symptoms and clinicians’ recording practices, not true biological differences.

“More classic symptoms…predominated” is not a fair comparison unless you explicitly acknowledge reporting bias.

The citations are fine, but the paragraph needs one explicit sentence acknowledging age-related symptom ascertainment limitations.

**Response: We thank the reviewer for this important clinical and methodological observation. We agree that the phrase “most relevant finding” is subjective and that direct symptom comparisons across age groups can be biased by age-related limitations in symptom reporting and differences in clinical documentation. We also agree that statements suggesting that “more classic symptoms predominated” should be accompanied by an explicit acknowledgement of potential reporting/ascertainment bias.**

**Action Taken: We revised the paragraph to remove value-judgment language, to describe the symptom patterns more neutrally, and to add an explicit sentence noting that subjective symptoms (e.g., headache, myalgia, chills) may be under-ascertained in children <5 years and influenced by documentation practices. We also clarified that these age-group differences should be interpreted cautiously in light of these limitations.**

**Location: Page 17-18**

**Page 17, Paragraph 4:** This paragraph is generally reasonable, but it still risks overgeneralization. The study includes only MAT-confirmed mild outpatient cases, so “clinical profile of children affected” should be narrowed to “in this primary care-based series”.

“May contribute to underreporting” is plausible but speculative; if retained, it should be clearly framed as a hypothesis.

Good applied recommendation (training), but make sure you avoid implying that training alone solves underdiagnosis; diagnostic access matters too.

**Response: We thank the reviewer for these helpful suggestions to avoid overgeneralization. We agree that our findings should be framed specifically as applying to MAT-confirmed, predominantly mild cases evaluated in primary care. We also agree that “may contribute to underreporting” should be presented as a hypothesis, and that recommendations should not imply training alone addresses underdiagnosis.**

**Action Taken: We revised the paragraph to explicitly limit the interpretation to this primary care based case series, reframed potential under-recognition as a hypothesis, and expanded the recommendation to emphasize both clinician training and access to diagnostic testing and referral pathways.**

**Location: Page 18**

**Page 17, Paragraph 5:** Methodological weakness is not acknowledged: BMI is not an optimal metric for <5; you should either justify its use or state it as a limitation before interpreting. Without that, the paragraph appears overconfident.

Causal speculation (“malnutrition compromises immune response and could influence the less florid presentation”). This is biologically plausible but not demonstrated by this dataset. It also risks circular reasoning and overreach.

Consider confounding: underweight is strongly correlated with poverty, sanitation, coinfections, none measured. The Discussion must acknowledge unmeasured confounding.

**Response: We thank the reviewer for this important comment. We agree that the previous wording was overly strong and could be interpreted as implying a causal relationship between undernutrition and clinical presentation that cannot be established from this retrospective descriptive study. We also agree that nutritional classification in children under 5 years of age must be interpreted using age-appropriate anthropometric standards and that potential unmeasured confounding should be explicitly acknowledged.**

**Action Taken: We revised the manuscript in three ways. First, we clarified in the Methods that nutritional status was classified using age-specific anthropometric criteria according to World Health Organization recommendations, including weight-for-height Z score (WHZ) for children under 5 years of age, rather than BMI alone. Second, we rewrote the Discussion paragraph to remove speculative or causal language regarding malnutrition, immune response, and symptom expression, and to frame the nutritional findings as descriptive and hypothesis-generating. Third, we added explicit acknowledgment in the Discussion/limitations that the observed differences in nutritional status may be influenced by unmeasured confounders not captured in the retrospective records, including poverty, sanitation conditions, coinfections, and barriers to care. Accordingly, we now avoid interpreting undernutrition as an independent explanatory factor in this dataset.**

**Location: Methods page 9 and Discussion Page 18**

**Page 17, Paragraph 6:** The descriptive observation (46% within 0–3 days) is fine, but the paragraph over-links it to clinical presentation differences in a way that reads causal. Also, Results/Methods have inconsistencies in how “duration” is analyzed (categorical vs continuous). The Discussion should not lean heavily on duration until the analysis is fixed. Keep as descriptive and explicitly mention potential bias.

**Response: We thank the reviewer for this important point. We agree that linking time to consultation with symptom differences can read as causal and that “duration” must be described consistently with the categorical approach used in our analyses.**

**Action Taken: We revised the paragraph to present timing of consultation and symptom frequencies descriptively without causal language, clarified that time from symptom onset to consultation was analyzed in categories, and added explicit caveats regarding caregiver recall/reporting and age-related differences in symptom ascertainment.**

**Location: Page 18**

**Page 17,** **Paragraph 7:** This is one of the paragraphs that most needs correction.

The Results/Table label these as “species,” but they are MAT serovars/serogroups. The Discussion should correct and use consistent terms.

“Panama serovar significantly more frequent (7% vs 1%)” corresponds to 2 vs 4 cases. That is extremely fragile and likely violates chi-square assumptions (Fisher would be appropriate). This must be explicitly acknowledged.

Claims about “virulence, transmission patterns, immunological susceptibility” are not supported by MAT data and tiny counts.

**Response: We thank the reviewer for noting the incorrect terminology and the overinterpretation of sparse MAT serovar/serogroup findings. We agree that these are MAT serovar/serogroup reactivity patterns, not “species,” and that very small cell counts require Fisher’s exact test and cautious interpretation.**

**Action Taken: We replaced the term “species” with “serovar/serogroup reactivity” throughout the manuscript and revised the Discussion to use consistent MAT terminology. We also clarified that the apparent difference in Panama reactivity was based on very small counts and should be interpreted with caution. In addition, we revised the text to avoid claims regarding virulence, transmission patterns, or immunological susceptibility that are not supported by MAT seroreactivity data.**

**Location: Page 18**

**Page 18, Paragraph 8:** The descriptive reporting is fine, but do not imply appropriateness without evidence. You do not assess outcomes, adherence, dosing, timing, or guideline concordance systematically. Some antibiotic differences are expected due to age-related contraindications (e.g., doxycycline). This should be stated explicitly. “Crucial to prevent complications” is true generally, but your dataset contains only mild cases and no outcomes. This sentence is a bit disconnected from the evidence.

**Response: We thank the reviewer for this important point and agree that our dataset does not allow assessment of appropriateness, guideline concordance, dosing, timing, adherence, or clinical outcomes.**

**Action Taken: We revised the paragraph to keep the reporting strictly descriptive, removed language implying appropriateness or prevention of complications, explicitly noted that treatment reflects routine outpatient practice influenced by age-related prescribing considerations, and clarified that outcomes and prescribing decision criteria were not available in this retrospective record review.**

**Location: Page 18-19**

**Page 18, Paragraph 9:** Consider strengthening it by explicitly stating: selection context: primary care only, referral mechanism, and how this affects generalizability to severe disease. Also add: lack of lab confirmation beyond MAT, and missingness concerns (if applicable).

**Response: We thank the reviewer for this helpful recommendation. We agree that the concluding paragraph should more explicitly describe the selection context of our primary care–based series and its implications for generalizability and clarify diagnostic and documentation constraint.**

**Action Taken: We strengthened the paragraph to explicitly state that our sample comprises MAT-confirmed cases evaluated in first-level outpatient facilities, that referral of more severe cases to higher levels of care may limit generalizability to severe/hospitalized leptospirosis, and that confirmation in our dataset relied on MAT with limited availability of additional laboratory confirmation in the clinical records. We also added a brief statement noting incomplete documentation for some variables and variable denominators across analyses.**

**Location: Page 19**

**Page 18, Paragraph 10:** This paragraph over-summarizes in a way that implies a stable “profile,” but many listed elements are not strongly supported:

- “predominance of female sex” with n=28 is weak and likely random.
- “rural or peri-urban origin” is descriptive but not tested as significant.
- “residence in flood-prone areas” differs only modestly (50% vs 43%).
- “low BMI” is methodologically questionable in <5.
- “Panama followed by Varillal” is based on tiny counts and MAT limitations.

The concluding policy recommendation (“justify the need to strengthen surveillance…”) is reasonable, but it should be framed as supported by the high-burden context and diagnostic challenges, not by fragile subgroup associations.

**Response: We thank the reviewer for this important observation. We agree that the previous paragraph over-summarized the findings and could be misread as implying a stable or definitive clinical “profile” for children under 5 years of age, despite the small pediatric sample and the exploratory nature of the comparisons. We also agree that several of the previously listed characteristics were descriptive only and should not be emphasized as robust subgroup findings.**

**Action Taken: We rewrote the concluding paragraph of the Discussion to remove overgeneralized statements regarding female sex, rural or peri-urban origin, flood-prone residence, low BMI, and Panama reactivity. The revised text now focuses more cautiously on the descriptive finding that young children commonly presented with nonspecific symptoms such as malaise and fever, while explicitly acknowledging the limited sample size and exploratory nature of the age-stratified comparisons. We also reframed the final implication regarding surveillance so that it is supported by the high-burden endemic context and the diagnostic challenges posed by nonspecific presentation in young children, rather than by fragile subgroup associations.**

**Location: Page 19**

# Limitations Paragraph

The current Limitations section is framed too narrowly and does not adequately reflect the full range of methodological and analytical constraints of the study. At present, the paragraph focuses almost exclusively on the absence of severe cases and deaths, which is an important point but represents only one dimension of the study’s limitations. Several additional factors that directly affect the interpretation and robustness of the findings are not acknowledged and should be explicitly discussed. In particular, the very small number of children under five years of age substantially limits statistical power for age-stratified comparisons, multivariate analyses, and serovar-specific associations, and this limitation should be clearly stated. The retrospective design based on medical record review introduces potential selection bias, as inclusion depended on the completeness of clinical records, as well as information bias related to variable documentation quality across patients, age groups, and health centers. Differential symptom ascertainment by age is another critical limitation that is not addressed; several symptoms compared between age groups rely on subjective reporting and may be under-recognized or under-recorded in young children, which could partly explain observed differences rather than true biological variation. In addition, diagnosis and serovar attribution relied exclusively on MAT performed on single serum samples, without paired sera, PCR, or culture confirmation; given the known cross-reactivity and limited resolution of MAT in endemic settings, this limitation is particularly relevant in light of the serovar-specific interpretations presented. The assessment of nutritional status in children under five using BMI should also be recognized as a limitation, as BMI is not the optimal anthropometric indicator for this age group and may lead to misclassification. Furthermore, the markedly unequal contribution of cases across participating health centers may reflect differences in healthcare access, testing practices, or catchment populations rather than true epidemiological variation, and this potential center-level bias should be acknowledged. Finally, the limitations section should clarify that multiple comparisons were performed in an exploratory context and that the findings are best interpreted as descriptive and hypothesis-generating rather than confirmatory.

**Response: We thank the reviewer for this thoughtful and comprehensive comment. We agree that the original limitations section was too narrow and did not sufficiently reflect the broader methodological and analytical constraints of the study.**

**Action Taken: We substantially revised the Limitations section to better reflect the full range of study constraints. Specifically, we added explicit discussion of the small number of children under 5 years of age and the resulting limited statistical power for subgroup comparisons; the potential for selection and information bias inherent to retrospective record review; differential symptom ascertainment by age, particularly for subjective symptoms in young children; the limitations of MAT-based classification without paired sera or other confirmatory methods; the marked imbalance in case contribution across health centers and the possibility of center-level ascertainment bias; and the exploratory nature of the multiple comparisons. We also clarified that nutritional findings in children under 5 years should be interpreted cautiously, noting that although age-appropriate anthropometric criteria were used, the retrospective records did not capture important potential confounders such as poverty, sanitation, and coinfections.**

**Location: Page 19-20**

**Language and Style**

Minor grammatical issues occur throughout (e.g., inconsistent pluralization, occasional awkward phrasing). A careful language revision by a fluent English speaker is recommended.

Consistency should be ensured for terms such as “under 5 years,” “under five,” and “<5 years.”

**Response: We thank the reviewer for this helpful comment. We agree that the manuscript required careful language revision to improve clarity, consistency, and readability.**

**Action Taken: We performed a thorough language and style revision throughout the manuscript to correct minor grammatical issues, improve phrasing, and standardize terminology. We adopted “under 5 years” consistently across the manuscript in place of mixed forms such as “under five,” “<5 years,” and related variants, except where mathematical notation was retained in tables or figure labels for space or formatting reasons.**

**Location: Throughout Manuscript**
