# Supplementary material for: Clinical and epidemiological characteristics of leptospirosis in patients under and over 5 years of age in primary health centers in the Peruvian Amazon, 2022–2024
Source: PLoS Negl Trop Dis. 2026 Jun 25;20(6):e0013473. doi: 10.1371/journal.pntd.0013473 (PMC13421768; doi:10.1371/journal.pntd.0013473)
Supplement: Peer review history

# Peer review: Decision Letter 0

**DOI:** 10.1371/journal.pntd.0013473.r001

**Authors:** Stuart D. Blacksell, Yung-Fu Chang

13 Jan 2026

PNTD-D-25-01422

Differential profile of Leptospirosis in patients under and over 5 years of age in health centers of the Peruvian Amazon, 2023–2024

PLOS Neglected Tropical Diseases

Dear Dr. Zevallos,

Thank you for submitting your manuscript to PLOS Neglected Tropical Diseases. After careful consideration, we feel that it has merit but does not fully meet PLOS Neglected Tropical Diseases's publication criteria as it currently stands. Therefore, we invite you to submit a revised version of the manuscript that addresses the points raised during the review process.

Please submit your revised manuscript within by Mar 14 2026 11:59PM. If you will need more time than this to complete your revisions, please reply to this message or contact the journal office at plosntds@plos.org. When you're ready to submit your revision, log on to https://www.editorialmanager.com/pntd/ and select the 'Submissions Needing Revision' folder to locate your manuscript file.

Please include the following items when submitting your revised manuscript:

* A letter that responds to each point raised by the editor and reviewer(s). You should upload this letter as a separate file labeled 'Response to Reviewers'. This file does not need to include responses to any formatting updates and technical items listed in the 'Journal Requirements' section below.

* A marked-up copy of your manuscript that highlights changes made to the original version. You should upload this as a separate file labeled 'Revised Manuscript with Track Changes'.

* An unmarked version of your revised paper without tracked changes. You should upload this as a separate file labeled 'Manuscript'.

If you would like to make changes to your financial disclosure, competing interests statement, or data availability statement, please make these updates within the submission form at the time of resubmission. Guidelines for resubmitting your figure files are available below the reviewer comments at the end of this letter.

We look forward to receiving your revised manuscript.

Kind regards,

Yung-Fu Chang

Academic Editor

PLOS Neglected Tropical Diseases

Stuart Blacksell

Section Editor

PLOS Neglected Tropical Diseases

Shaden Kamhawi

co-Editor-in-Chief

PLOS Neglected Tropical Diseases

orcid.org/0000-0003-4304-636XX

Paul Brindley

co-Editor-in-Chief

PLOS Neglected Tropical Diseases

orcid.org/0000-0003-1765-0002

**Additional Editor Comments:**

1. The author states that “A case was considered confirmed when a single blood sample showed a serological titer equal to or greater than 1:800, also identifying the serological variant.” Please provide justification, preferably with a citation, why this MAT titer was selected.
2. State the Leptospira serovars that were used in the MAT in the methods section. At the moment, these are detailed in Table 4, but it would be helpful to have them detailed in the methods as well.
3. How many patients had paired samples?
4. Table 4 indicates that some patients were MAT-positive against 2, 3, or 4 serovars. Is this multiple positivity or cross-reactivity? Do you have the actual titers that could demonstrate superiority or the equivalence of a particular serovar in such a case?

**Journal Requirements:**

If the reviewer comments include a recommendation to cite specific previously published works, please review and evaluate these publications to determine whether they are relevant and should be cited. There is no requirement to cite these works unless the editor has indicated otherwise.

1) Please upload all main figures as separate Figure files in .tif or .eps format. For more information about how to convert and format your figure files please see our guidelines:

https://journals.plos.org/plosntds/s/figures

2) We have noticed that you have uploaded Supporting Information files, but you have not included a list of legends. Please add a full list of legends for your Supporting Information files after the references list.

3) Some material included in your submission may be copyrighted. According to PLOSu2019s copyright policy, authors who use figures or other material (e.g., graphics, clipart, maps) from another author or copyright holder must demonstrate or obtain permission to publish this material under the Creative Commons Attribution 4.0 International (CC BY 4.0) License used by PLOS journals. Please closely review the details of PLOSu2019s copyright requirements here: PLOS Licenses and Copyright. If you need to request permissions from a copyright holder, you may use PLOS's Copyright Content Permission form.

Please respond directly to this email and provide any known details concerning your material's license terms and permissions required for reuse, even if you have not yet obtained copyright permissions or are unsure of your material's copyright compatibility. Once you have responded and addressed all other outstanding technical requirements, you may resubmit your manuscript within Editorial Manager.

Potential Copyright Issues:

i) Figure 2. Please (a) provide a direct link to the base layer of the map (i.e., the country or region border shape) and ensure this is also included in the figure legend; and (b) provide a link to the terms of use / license information for the base layer image or shapefile. We cannot publish proprietary or copyrighted maps (e.g. Google Maps, Mapquest) and the terms of use for your map base layer must be compatible with our CC BY 4.0 license.

Note: if you created the map in a software program like R or ArcGIS, please locate and indicate the source of the basemap shapefile onto which data has been plotted.

If your map was obtained from a copyrighted source please amend the figure so that the base map used is from an openly available source. Alternatively, please provide explicit written permission from the copyright holder granting you the right to publish the material under our CC BY 4.0 license.

If you are unsure whether you can use a map or not, please do reach out and we will be able to help you. The following websites are good examples of where you can source open access or public domain maps:

* U.S. Geological Survey (USGS) - All maps are in the public domain. (http://www.usgs.gov)

* PlaniGlobe - All maps are published under a Creative Commons license so please cite u201cPlaniGlobe, http://www.planiglobe.com, CC BY 2.0u201d in the image credit after the caption. (http://www.planiglobe.com/?lang=enl)

* Natural Earth - All maps are public domain. (http://www.naturalearthdata.com/about/terms-of-use/).

**Reviewers' Comments:**

Reviewer's Responses to Questions

**Key Review Criteria Required for Acceptance?**

As you describe the new analyses required for acceptance, please consider the following:

**Methods**

-Are the objectives of the study clearly articulated with a clear testable hypothesis stated?

-Is the study design appropriate to address the stated objectives?

-Is the population clearly described and appropriate for the hypothesis being tested?

-Is the sample size sufficient to ensure adequate power to address the hypothesis being tested?

-Were correct statistical analysis used to support conclusions?

-Are there concerns about ethical or regulatory requirements being met?

Reviewer #1: Are the objectives of the study clearly articulated with a clear testable hypothesis stated?

The objectives are generally stated and descriptive in nature; however, a clear, testable hypothesis is not explicitly articulated, and some analyses imply hypothesis testing beyond the stated descriptive aim.

Is the study design appropriate to address the stated objectives?

The retrospective, primary care–based design is appropriate for descriptive purposes but is not sufficient to support some of the inferential and multivariate conclusions currently presented.

Is the population clearly described and appropriate for the hypothesis being tested?

The study population is clearly described and relevant to the research question; however, the very small number of children under five limits the appropriateness of age-stratified inferential analyses.

Is the sample size sufficient to ensure adequate power to address the hypothesis being tested?

No. While the overall sample is reasonable, the pediatric subgroup (<5 years) is underpowered for robust statistical comparisons, multivariate modeling, and serovar-specific analyses.

Were correct statistical analyses used to support conclusions?

Several statistical concerns remain, including inappropriate or unstable multivariate modeling, use of chi-square tests with sparse data, lack of adjustment for multiple comparisons, and inconsistencies in variable handling. Reanalysis is required.

Are there concerns about ethical or regulatory requirements being met?

No major ethical concerns were identified; appropriate ethical approval is reported for retrospective use of anonymized clinical records.

**********

**Results**

-Does the analysis presented match the analysis plan?

-Are the results clearly and completely presented?

-Are the figures (Tables, Images) of sufficient quality for clarity?

Reviewer #1: Does the analysis presented match the analysis plan?

Not fully. There are inconsistencies between the described analysis plan and the analyses presented, particularly regarding the handling of key variables (e.g., duration of illness) and the use and interpretation of multivariate regression.

Are the results clearly and completely presented?

The results are generally well organized and descriptive; however, several findings are overinterpreted, some statistical comparisons lack sufficient context (e.g., sparse cell counts, multiple testing), and key clarifications are needed to ensure internal consistency and transparency.

Are the figures (Tables, Images) of sufficient quality for clarity?

Tables are generally clear, but at least one figure (the study area map) does not meet scientific publication standards and should be replaced with a properly generated GIS-based figure.

**********

**Conclusions**

-Are the conclusions supported by the data presented?

-Are the limitations of analysis clearly described?

-Do the authors discuss how these data can be helpful to advance our understanding of the topic under study?

-Is public health relevance addressed?

Reviewer #1: Are the conclusions supported by the data presented?

Partially. While the descriptive findings are supported by the data, several conclusions—particularly those based on multivariate analyses and serovar-specific associations—currently exceed what the data can robustly support.

Are the limitations of analysis clearly described?

No. The limitations section is incomplete and does not adequately address key methodological, statistical, and diagnostic constraints that affect interpretation.

Do the authors discuss how these data can be helpful to advance our understanding of the topic under study?

Yes, in a general sense, particularly regarding outpatient presentations in a hyperendemic setting; however, this contribution would be clearer if interpretations were more restrained and aligned with the descriptive nature of the study.

Is public health relevance addressed?

Yes. The manuscript clearly highlights public health relevance, especially for primary care surveillance and early recognition in endemic regions, although some implications should be framed more cautiously.

**********

**Editorial and Data Presentation Modifications?**

Use this section for editorial suggestions as well as relatively minor modifications of existing data that would enhance clarity. If the only modifications needed are minor and/or editorial, you may wish to recommend “Minor Revision” or “Accept”.

Reviewer #1: Several editorial and data presentation improvements are required to enhance clarity and scientific rigor. Terminology should be standardized throughout the manuscript (e.g., correct use of “serovar/serogroup” instead of “species,” consistent age-group labeling, and accurate use of epidemiological terms such as “case counts” rather than “prevalence”). Tables should consistently report absolute numbers alongside percentages, and reference categories should be clearly stated for all statistical comparisons. The Results section would benefit from more restrained language, clearly distinguishing descriptive findings from exploratory analyses. Figure quality also requires improvement, particularly the study area map, which should be replaced with a properly generated GIS-based figure meeting scientific publication standards. Finally, the Limitations section should be expanded to transparently address methodological, statistical, and diagnostic constraints. These changes, while partly editorial, are essential for accurate interpretation and should accompany the major analytical revisions requested; therefore, the required modifications go beyond minor or purely editorial revision.

**********

**Summary and General Comments**

Use this section to provide overall comments, discuss strengths/weaknesses of the study, novelty, significance, general execution and scholarship. You may also include additional comments for the author, including concerns about dual publication, research ethics, or publication ethics. If requesting major revision, please articulate the new experiments that are needed.

Reviewer #1: Please see the attached document.

**********

PLOS authors have the option to publish the peer review history of their article (what does this mean?). If published, this will include your full peer review and any attached files.

If you choose “no”, your identity will remain anonymous but your review may still be made public.

**Do you want your identity to be public for this peer review?**  For information about this choice, including consent withdrawal, please see our Privacy Policy.

Reviewer #1: No

[NOTE: If reviewer comments were submitted as an attachment file, they will be attached to this email and accessible via the submission site. Please log into your account, locate the manuscript record, and check for the action link "View Attachments". If this link does not appear, there are no attachment files.]

**Figure resubmission:**

While revising your submission, we strongly recommend that you use PLOS’s NAAS tool (https://ngplosjournals.pagemajik.ai/artanalysis) to test your figure files. NAAS can convert your figure files to the TIFF file type and meet basic requirements (such as print size, resolution), or provide you with a report on issues that do not meet our requirements and that NAAS cannot fix.

After uploading your figures to PLOS’s NAAS tool - https://ngplosjournals.pagemajik.ai/artanalysis, NAAS will process the files provided and display the results in the "Uploaded Files" section of the page as the processing is complete. If the uploaded figures meet our requirements (or NAAS is able to fix the files to meet our requirements), the figure will be marked as "fixed" above. If NAAS is unable to fix the files, a red "failed" label will appear above. When NAAS has confirmed that the figure files meet our requirements, please download the file via the download option, and include these NAAS processed figure files when submitting your revised manuscript.

**Reproducibility:**

To enhance the reproducibility of your results, we recommend that authors of applicable studies deposit laboratory protocols in protocols.io, where a protocol can be assigned its own identifier (DOI) such that it can be cited independently in the future. Additionally, PLOS ONE offers an option to publish peer-reviewed clinical study protocols. Read more information on sharing protocols at https://plos.org/protocols?utm_medium=editorial-email&utm_source=authorletters&utm_campaign=protocols

Attachment Submitted filename: My Comments_PNTD-D-25-01422.pdf

# Author comment: Author response to Decision Letter 1

**DOI:** 10.1371/journal.pntd.0013473.r002

1 Apr 2026

Attachment Submitted filename: Comments_PNTD-D-25-01422 REPLY TO COMMENTS 13 March 2026.docx

# Peer review: Decision Letter 1

**DOI:** 10.1371/journal.pntd.0013473.r003

**Authors:** Stuart D. Blacksell, Yung-Fu Chang

28 Apr 2026

PNTD-D-25-01422R1

Differential profile of Leptospirosis in patients under and over 5 years of age in health centers of the Peruvian Amazon, 2022–2024

PLOS Neglected Tropical Diseases

Dear Dr. Zevallos,

Thank you for submitting your manuscript to PLOS Neglected Tropical Diseases. After careful consideration, we feel that it has merit but does not fully meet PLOS Neglected Tropical Diseases's publication criteria as it currently stands. Therefore, we invite you to submit a revised version of the manuscript that addresses the points raised during the review process.

Please submit your revised manuscript by May 28 2026 11:59PM. If you will need more time than this to complete your revisions, please reply to this message or contact the journal office at plosntds@plos.org. When you're ready to submit your revision, log on to https://www.editorialmanager.com/pntd/ and select the 'Submissions Needing Revision' folder to locate your manuscript file.

Please include the following items when submitting your revised manuscript:

* A letter that responds to each point raised by the editor and reviewer(s). You should upload this letter as a separate file labeled 'Response to Reviewers'. This file does not need to include responses to any formatting updates and technical items listed in the 'Journal Requirements' section below.

* A marked-up copy of your manuscript that highlights changes made to the original version. You should upload this as a separate file labeled 'Revised Manuscript with Track Changes'.

* An unmarked version of your revised paper without tracked changes. You should upload this as a separate file labeled 'Manuscript'.

If you would like to make changes to your financial disclosure, competing interests statement, or data availability statement, please make these updates within the submission form at the time of resubmission. Guidelines for resubmitting your figure files are available below the reviewer comments at the end of this letter.

We look forward to receiving your revised manuscript.

Kind regards,

Yung-Fu Chang

Academic Editor

PLOS Neglected Tropical Diseases

Stuart Blacksell

Section Editor

PLOS Neglected Tropical Diseases

Shaden Kamhawi

co-Editor-in-Chief

PLOS Neglected Tropical Diseases

orcid.org/0000-0003-4304-636XX

Paul Brindley

co-Editor-in-Chief

PLOS Neglected Tropical Diseases

orcid.org/0000-0003-1765-0002

**Journal Requirements:**

If the reviewer comments include a recommendation to cite specific previously published works, please review and evaluate these publications to determine whether they are relevant and should be cited. There is no requirement to cite these works unless the editor has indicated otherwise.

1) Your manuscript's sections are not in the correct order.  Please amend to the following order: Abstract, Introduction, Results, Discussion, and Methods

**Reviewers' comments:**

Reviewer's Responses to Questions

**Key Review Criteria Required for Acceptance?**

As you describe the new analyses required for acceptance, please consider the following:

**Methods**

-Are the objectives of the study clearly articulated with a clear testable hypothesis stated?

-Is the study design appropriate to address the stated objectives?

-Is the population clearly described and appropriate for the hypothesis being tested?

-Is the sample size sufficient to ensure adequate power to address the hypothesis being tested?

-Were correct statistical analysis used to support conclusions?

-Are there concerns about ethical or regulatory requirements being met?

Reviewer #1: The objective is now clearly stated and appropriately framed as descriptive and comparative; however, a formal testable hypothesis is still not explicitly defined. The study is better positioned as exploratory rather than hypothesis-driven.

Yes. The retrospective cross-sectional design is appropriate for the stated descriptive objective of characterizing clinical and epidemiological patterns in a primary care setting, although it remains limited for causal inference.

Yes. The study population is now clearly defined, including the total suspected cases and selection process, and is appropriate for the descriptive aims. However, representativeness is influenced by primary care–based sampling and uneven distribution across centers.

No. While the overall sample size (n=400) is adequate for descriptive purposes, the subgroup of children under 5 years (n=28) remains underpowered for robust comparative or inferential analyses.

Partially. The statistical approach has improved, including the use of Fisher’s exact test and explicit acknowledgment of exploratory analyses without multiple testing correction. However, results should still be interpreted cautiously given small cell counts, multiple comparisons, and limited power.

No. Ethical approval, data anonymization, and waiver of informed consent are clearly described and appropriate for a retrospective study using routine clinical records.

**********

**Results**

-Does the analysis presented match the analysis plan?

-Are the results clearly and completely presented?

-Are the figures (Tables, Images) of sufficient quality for clarity?

Reviewer #1: Yes, overall. The analyses presented are now consistent with the stated plan, including descriptive summaries and exploratory bivariate comparisons. The authors appropriately clarified that comparisons are exploratory and avoided overuse of multivariate modeling.

Yes, for the most part. The results are clearly structured, with appropriate separation between descriptive findings and exploratory comparisons. The inclusion of supplementary tables improves transparency. However, interpretation should remain cautious given the small pediatric sample and exploratory nature of the analyses.

Tables are clear and informative. Figures have improved (e.g., inclusion of a GIS-based map), but some visual elements, particularly the density map, could still be refined to better align with scientific cartographic standards and avoid potential misinterpretation.

**********

**Conclusions**

-Are the conclusions supported by the data presented?

-Are the limitations of analysis clearly described?

-Do the authors discuss how these data can be helpful to advance our understanding of the topic under study?

-Is public health relevance addressed?

Reviewer #1: Yes, largely. The conclusions are now more appropriately aligned with the descriptive and exploratory nature of the study, and major overinterpretations have been addressed.

Yes. The limitations section has been substantially improved and now addresses key methodological, statistical, and contextual constraints, although a few additional points could still be more explicitly stated.

Yes. The manuscript clearly highlights the relevance of outpatient and pediatric presentations in a hyperendemic setting and frames the findings as contributing to improved clinical awareness and future research directions.

Yes. The study effectively emphasizes implications for primary care recognition, surveillance, and management of leptospirosis in vulnerable populations within endemic regions.

**********

**Editorial and Data Presentation Modifications?**

Use this section for editorial suggestions as well as relatively minor modifications of existing data that would enhance clarity. If the only modifications needed are minor and/or editorial, you may wish to recommend “Minor Revision” or “Accept”.

Reviewer #1: The manuscript has been substantially improved in clarity, structure, and alignment between methods, results, and conclusions. Remaining issues are mostly minor and editorial. Terminology should be fully standardized throughout the text (e.g., consistent use of “serovar/serogroup” and uniform age-group definitions). Some sentences could benefit from minor language polishing to improve readability and precision in English. Tables are generally clear, but minor formatting adjustments (e.g., consistent reporting of percentages and reference categories) would further enhance clarity. The revised figures represent an improvement, particularly the inclusion of a GIS-based map; however, minor refinements in cartographic presentation (e.g., legend clarity and symbology) could still improve interpretability. Overall, only minor editorial adjustments are needed to finalize the manuscript for publication.

**********

**Summary and General Comments**

Use this section to provide overall comments, discuss strengths/weaknesses of the study, novelty, significance, general execution and scholarship. You may also include additional comments for the author, including concerns about dual publication, research ethics, or publication ethics. If requesting major revision, please articulate the new experiments that are needed.

Reviewer #1: The revised version shows substantial improvement compared to the original submission. The authors have appropriately reframed the study as descriptive and exploratory, clarified the study population and denominators, improved the statistical approach (including the use of Fisher’s exact test and clearer distinction between descriptive and inferential analyses), and significantly strengthened the Limitations section. Importantly, conclusions are now more proportionate to the data and avoid prior overinterpretation.

The main strengths of the study include the use of laboratory-confirmed cases, the multi-year dataset, and the focus on primary care settings, which are often underrepresented in leptospirosis research. The emphasis on young children, despite the small sample size, highlights an important gap in the literature and provides a basis for future investigation.

Remaining weaknesses are relatively minor and largely editorial. These include the need for full standardization of terminology, minor improvements in clarity and English language, and small refinements in table formatting and figure presentation. While the limitations have been substantially improved, a few additional points (e.g., symptom ascertainment bias in young children and diagnostic constraints related to MAT) could be stated more explicitly, although these do not materially compromise the overall interpretation.

I suggest revising the title to better reflect the descriptive and exploratory nature of the study and to avoid implying strong differential patterns that are not fully supported by the data. A more neutral and precise formulation would improve alignment between the title and the study design. My suggestion: "Clinical and epidemiological characteristics of leptospirosis in patients under and over 5 years of age in primary health centers in the Peruvian Amazon, 2022–2024"

**********

PLOS authors have the option to publish the peer review history of their article (what does this mean?). If published, this will include your full peer review and any attached files.

If you choose “no”, your identity will remain anonymous but your review may still be made public.

**Do you want your identity to be public for this peer review?**  For information about this choice, including consent withdrawal, please see our Privacy Policy.

Reviewer #1: No

[NOTE: If reviewer comments were submitted as an attachment file, they will be attached to this email and accessible via the submission site. Please log into your account, locate the manuscript record, and check for the action link "View Attachments". If this link does not appear, there are no attachment files.]

**Figure resubmission:**

While revising your submission, we strongly recommend that you use PLOS’s NAAS tool (https://ngplosjournals.pagemajik.ai/artanalysis) to test your figure files. NAAS can convert your figure files to the TIFF file type and meet basic requirements (such as print size, resolution), or provide you with a report on issues that do not meet our requirements and that NAAS cannot fix.

After uploading your figures to PLOS’s NAAS tool - https://ngplosjournals.pagemajik.ai/artanalysis, NAAS will process the files provided and display the results in the "Uploaded Files" section of the page as the processing is complete. If the uploaded figures meet our requirements (or NAAS is able to fix the files to meet our requirements), the figure will be marked as "fixed" above. If NAAS is unable to fix the files, a red "failed" label will appear above. When NAAS has confirmed that the figure files meet our requirements, please download the file via the download option, and include these NAAS processed figure files when submitting your revised manuscript.

**Reproducibility:**

To enhance the reproducibility of your results, we recommend that authors of applicable studies deposit laboratory protocols in protocols.io, where a protocol can be assigned its own identifier (DOI) such that it can be cited independently in the future. Additionally, PLOS ONE offers an option to publish peer-reviewed clinical study protocols. Read more information on sharing protocols at https://plos.org/protocols?utm_medium=editorial-email&utm_source=authorletters&utm_campaign=protocols

# Author comment: Author response to Decision Letter 2

**DOI:** 10.1371/journal.pntd.0013473.r004

3 Jun 2026

Attachment Submitted filename: Response to Reviewers - May 27 2026.docx

# Editor report: Decision Letter 2

**DOI:** 10.1371/journal.pntd.0013473.r005

**Authors:** Stuart D. Blacksell, Yung-Fu Chang

5 Jun 2026

Dear Dr Zevallos,

We are pleased to inform you that your manuscript 'Clinical and epidemiological characteristics of leptospirosis in patients under and over 5 years of age in primary health centers in the Peruvian Amazon, 2022–2024' has been provisionally accepted for publication in PLOS Neglected Tropical Diseases.

Before your manuscript can be formally accepted you will need to complete some formatting changes, which you will receive in a follow up email. A member of our team will be in touch with a set of requests.

Please note that your manuscript will not be scheduled for publication until you have made the required changes, so a swift response is appreciated.

IMPORTANT: The editorial review process is now complete. PLOS will only permit corrections to spelling, formatting or significant scientific errors from this point onwards. Requests for major changes, or any which affect the scientific understanding of your work, will cause delays to the publication date of your manuscript.

Should you, your institution's press office or the journal office choose to press release your paper, you will automatically be opted out of early publication. We ask that you notify us now if you or your institution is planning to press release the article. All press must be co-ordinated with PLOS.

Thank you again for supporting Open Access publishing; we are looking forward to publishing your work in PLOS Neglected Tropical Diseases.

Best regards,

Yung-Fu Chang

Academic Editor

PLOS Neglected Tropical Diseases

Stuart Blacksell

Section Editor

PLOS Neglected Tropical Diseases

Shaden Kamhawi

co-Editor-in-Chief

PLOS Neglected Tropical Diseases

orcid.org/0000-0003-4304-636XX

Paul Brindley

co-Editor-in-Chief

PLOS Neglected Tropical Diseases

orcid.org/0000-0003-1765-0002

***********************************************************

p.p1 {margin: 0.0px 0.0px 0.0px 0.0px; line-height: 16.0px; font: 14.0px Arial; color: #323333; -webkit-text-stroke: #323333}span.s1 {font-kerning: none

# Editor report: Acceptance letter

**DOI:** 10.1371/journal.pntd.0013473.r006

**Authors:** Stuart D. Blacksell, Yung-Fu Chang

Dear Dr Zevallos,

We are delighted to inform you that your manuscript, "Clinical and epidemiological characteristics of leptospirosis in patients under and over 5 years of age in primary health centers in the Peruvian Amazon, 2022–2024," has been formally accepted for publication in PLOS Neglected Tropical Diseases.

We have now passed your article onto the PLOS Production Department who will complete the rest of the publication process. All authors will receive a confirmation email upon publication.

The corresponding author will soon be receiving a typeset proof for review, to ensure errors have not been introduced during production. Please review the PDF proof of your manuscript carefully, as this is the last chance to correct any scientific or type-setting errors. Please note that major changes, or those which affect the scientific understanding of the work, will likely cause delays to the publication date of your manuscript. Note: Proofs for Front Matter articles (Editorial, Viewpoint, Symposium, Review, etc...) are generated on a different schedule and may not be made available as quickly.

Soon after your final files are uploaded, the early version of your manuscript will be published online unless you opted out of this process. The date of the early version will be your article's publication date. The final article will be published to the same URL, and all versions of the paper will be accessible to readers.

For Research Articles, you will receive an invoice from PLOS for your publication fee after your manuscript has reached the completed accept phase. If you receive an email requesting payment before acceptance or for any other service, this may be a phishing scheme. Learn how to identify phishing emails and protect your accounts at https://explore.plos.org/phishing.

Thank you again for supporting open-access publishing; we are looking forward to publishing your work in PLOS Neglected Tropical Diseases.

Best regards,

Shaden Kamhawi

co-Editor-in-Chief

PLOS Neglected Tropical Diseases

Paul Brindley

co-Editor-in-Chief

PLOS Neglected Tropical Diseases
